# Nanocellulose-Based Passivated-Carbon Quantum Dots (P-CQDs) for Antimicrobial Applications: A Practical Review

**DOI:** 10.3390/polym15122660

**Published:** 2023-06-12

**Authors:** Sherif S. Hindi, Jamal S. M. Sabir, Uthman M. Dawoud, Iqbal M. Ismail, Khalid A. Asiry, Zohair M. Mirdad, Kamal A. Abo-Elyousr, Mohamed H. Shiboob, Mohamed A. Gabal, Mona Othman I. Albureikan, Rakan A. Alanazi, Omer H. M. Ibrahim

**Affiliations:** 1 Department of Agriculture, Faculty of Environmental Sciences, King Abdullaziz University (KAU), P.O. Box 80208, Jeddah 21589, Saudi Arabiaralanazi0010@stu.kau.edu.sa (R.A.A.); omerhoooo@gmail.com (O.H.M.I.); 2 Department of Biological Sciences, Faculty of Sciences, King Abdullaziz University (KAU), P.O. Box 80208, Jeddah 21589, Saudi Arabia; 3 Department of Chemical and Materials Engineering, King Abdullaziz University (KAU), P.O. Box 80208, Jeddah 21589, Saudi Arabia; 4 Department of Chemistry, Faculty of Science, Center of Excellence in Environmental Studies, King Abdullaziz University (KAU), P.O. Box 80208, Jeddah 21589, Saudi Arabia; 5 Plant Pathology Department, Faculty of Agriculture, Assiut University, Assiut 71526, Egypt; 6 Department of Environment, Faculty of Environmental Sciences, King Abdullaziz University (KAU), P.O. Box 80208, Jeddah 21589, Saudi Arabia; 7 Department of Chemistry, Faculty of Science, King Abdullaziz University (KAU), P.O. Box 80208, Jeddah 21589, Saudi Arabia

**Keywords:** microcrystalline cellulose, nanocrystalline cellulose, carbon quantum dots, functionalization, passivation, antiviral therapy, norovirus

## Abstract

Passivated-carbon quantum dots (P-CQDs) have been attracting great interest as an antimicrobial therapy tool due to their bright fluorescence, lack of toxicity, eco-friendly nature, simple synthetic schemes, and possession of photocatalytic functions comparable to those present in traditional nanometric semiconductors. Besides synthetic precursors, CQDs can be synthesized from a plethora of natural resources including microcrystalline cellulose (MCC) and nanocrystalline cellulose (NCC). Converting MCC into NCC is performed chemically via the top-down route, while synthesizing CODs from NCC can be performed via the bottom-up route. Due to the good surface charge status with the NCC precursor, we focused in this review on synthesizing CQDs from nanocelluloses (MCC and NCC) since they could become a potential source for fabricating carbon quantum dots that are affected by pyrolysis temperature. There are several P-CQDs synthesized with a wide spectrum of featured properties, namely functionalized carbon quantum dots (F-CQDs) and passivated carbon quantum dots (P-CQDs). There are two different important P-CQDs, namely 2,2′-ethylenedioxy-bis-ethylamine (EDA-CQDs) and 3-ethoxypropylamine (EPA-CQDs), that have achieved desirable results in the antiviral therapy field. Since NoV is the most common dangerous cause of nonbacterial, acute gastroenteritis outbreaks worldwide, this review deals with NoV in detail. The surficial charge status (SCS) of the P-CQDs plays an important role in their interactions with NoVs. The EDA-CQDs were found to be more effective than EPA-CQDs in inhibiting the NoV binding. This difference may be attributed to their SCS as well as the virus surface. EDA-CQDs with surficial terminal amino (-NH_2_) groups are positively charged at physiological pH (-NH^3+^), whereas EPA-CQDs with surficial terminal methyl groups (-CH_3_) are not charged. Since the NoV particles are negatively charged, they are attracted to the positively charged EDA-CQDs, resulting in enhancing the P-CQDs concentration around the virus particles. The carbon nanotubes (CNTs) were found to be comparable to the P-CQDs in the non-specific binding with NoV capsid proteins, through complementary charges, π-π stacking, and/or hydrophobic interactions.

## 1. Introduction

### 1.1. Nanocelluloses (NCs)

The most prevalent renewable organic substance on Earth is cellulose [1,2,3,4]. It can be extracted from plants, algae, and bacteria. Higher plants have primary and secondary cell walls that are made up of cellulose, hemicelluloses, lignin, and pectin. The distinctions between primary and secondary cell walls in terms of chemical make-up and structure are what give rise to the plant kingdom’s variety [1].

Several distinct types of nanoscale cellulosic fillers are possible due to the hierarchical and multilevel structure of cellulose. In addition to its nanocrystalline forms, cellulose also exists in an amorphous state that is randomly arranged in a spaghetti-like configuration, giving it a lower density. On the other hand, because they are vulnerable to intense acid attack, amorphous parts can be eliminated while leaving crystalline regions intact under certain circumstances [1,3,5].

**Figure 1:**
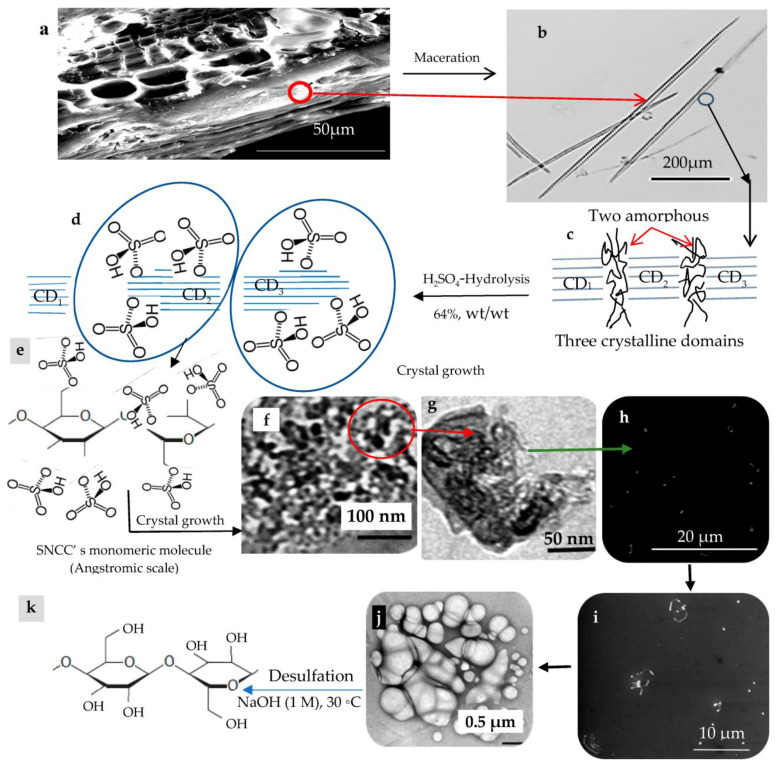
Formation of sulphated nanocrystalline cellulose (SNCCs): (**a**) SEM micrograph of anatomical structure of a typical wood tissue. (**b**) An optical image of macerated fibers. (**c**) The crystalline and amorphous domains within a microfibril. (**d**) SNCCs crystallite grafted by sulphated groups. (**e**) A monomeric molecule of SNCC. (**f**) TEM micrographs of SNCCs colony, and (**g**) Close-up image the SNCCs colony. (**h**) SEM micrographs of spreading and converging of the SCMCs. (**i**) A single colony with wider particles due to agglomeration. (**j**) SCMCs aggregation of single and multiball-shaped microcrystalline cellulose (SMCCs). (**k**) Desulphated cellobiose unit.

As shown in Figure 1, the anatomical structure of a typical wood tissue is clear (Figure 1a), besides showing some macerated fibers (MFs), as presented at Figure 1b, which are the famous natural resource of the cellulose precursor for the MCCs and NCCs products. Furthermore, cellulosic microfibrils are confirmed to be a consequence of crystalline and amorphous regions.

Cellulose-rich sources such as wood contain amorphous regions (Figure 1c) of cellulosic microfibrils that are degraded by acid hydrolysis to produce highly crystalline nanoparticles. Self-organization into a chiral nematic (cholesteric) liquid crystal phase with a helical configuration is a remarkable feature of NCCs. With the help of this remarkable property, dried NCC film can be utilized for security documents, mirrorless lasing, and liquid crystal displays (LCDs and LEDs). Size, dimensions, and other NCCs’ geometrical properties are also influenced by the composition of the cellulose precursors [2,3,4,5,6,7,8,9,10,11].

The amorphous regions are less dense than the crystalline domains and are constructed in a random manner like a spaghetti pattern (Figure 1c). As a result, the crystalline regions may remain unharmed while the amorphous regions are vulnerable to acid attack. Depending on their precursors, the majority of cellulosic materials contain crystalline and amorphous areas in varying proportions. The way that the cellulose molecules are organized has a significant impact on the physicochemical characteristics of the material. The majority of chemical reagents can only enter amorphous regions and can interact with crystallite surfaces [3,5] to create MCC and/or NCCs (Figure 1d–k).

The MCC is a partially hydrolyzed cellulose [2,4]. It can be obtained industrially from wood or lignocellulosic residues including linters, flosses, stalks, straw, rags, or shells of agricultural crops. The MCC is favorable in pharmaceutical, food, and cosmetic industries due to its high content of crystalline domains of the cellulosic microfibrils [2]. The MCC is one of the most important tableting excipients due to its outstanding dry binding properties of tablets for direct compression.

The nanometer range encompasses sizes larger than a few atoms and smaller than the visible light spectrum [4,11]. Due to their distinct mechanical characteristics, chirality, sustainability, and accessibility, colloidal NCCs rods with high aspect ratio (100–250 in length and 4–10 nm in width) have gained significant popularity in international markets [3,5,11].

Illustrating the large scale of the NCCs noticed in Figure 1, it is arisen from a so-called novel crystallographic phenomenon termed as crystal growth (Figure 1a–j). When NCC particles are approaching each other in an acidic aqueous atmosphere at a relatively warm temperature condition, they are susceptible to agglomerating electrostatically up to microscale particles, termed as pseudo-microcrystalline cellulose (PMCCs), which differ from ordinary MCCs in terms of their origin. For more illustration, the PMCC is agglomerated directly from NCC upon its crystal growth, while the ordinary MCCs are ingrained directly from cellulosic microfibrils harvested from plant’s cell wall. Despite both PMCCs and MCCs being situated within the microscale zone, they differ in their internal construction, especially crystallographic properties, namely crystallinity index (CI), crystallite size (CS), and lattice spacing (LS). It is worth mentioning that the NCCs have higher CI and CS, and lower LS than the MCCs, as examined by XRD.

For the sulphate groups (Figure 1e), grafted as a result of the acid hydrolysis of cellulosic microfibrils or MCC using sulfuric acid, we think that these functional groups may play an essential role in the agglomeration (upon crystal growth) and dissociation of micrometric particles (upon ingraining the NCCs from MCCs). Before synthesizing the CQDs from the SNCCs, they are desulphated using sodium hydroxide, as is seen in Figure 1k [12].

The nature of the cellulose precursors as well as the hydrolysis circumstances, such as duration, temperature, ultrasound treatment, and material purity, affect the geometrical properties of the NCCs, such as size, dimensions, and form [13,14,15]. The rod-like structure of the charged NCCs creates an anisotropic liquid crystalline phase above a critical concentration [4].

For the medicinal applications of the NCs, cellulose nanocrystals have the potential to be cutting-edge nanomaterials, according to Marpongahtun, et al. [11]. Due to their exceptional qualities, including good mechanical capabilities, low density, and an inherent renewable nature, nanocelluloses have gained a lot of attention in recent years [7]. These qualities make them ideal candidates for use as reinforcing nanofillers for various polymers. Additionally, CNCs have a number of benefits as starting materials for the creation of carbon structures, including a high fixed carbon content, low cost, and the exceptional ability to assemble into various morphologies (such as single nanoparticles, films, filaments, or aggregates). Then, specific carbon structures can be created by thermally decomposing these various CNC assemblies [16,17,18,19,20,21].

### 1.2. CQDs

The CQDs are small carbon nanoparticles (less than 10 nm in size) with some form of surficial passivation [22,23,24]. They possess the following properties: brightly fluorescent, non-toxic, ecofriendly, made with simple synthetic techniques, and have photocatalytic skills comparable to those of nanoscale semiconductors [11,25,26,27]. They have also attracted a lot of attention because of their stable photoluminescence properties, wide ranges of excitation and emission spectra, excellent biocompatibility, and little cytotoxicity effects on biological components. C-Dots are crucial in a number of applications [11]. The chemical modification of CQDs by adding organic molecules to their surfaces has created a novel class of materials with unique characteristics [23,28]. Valuable applications cover chemical and biological sensing, bioimaging, nanomedicine, photocatalysis, and electrocatalysis [25]. Among their unique properties is also their photo-catalytic antimicrobial function [27,29]. The CQDs with visible light illumination were found to be highly effective in inhibiting *Escherichia coli* cells, which can be attributed to their photodynamic effect [30].

### 1.3. P-CQDs

**Figure 2:**
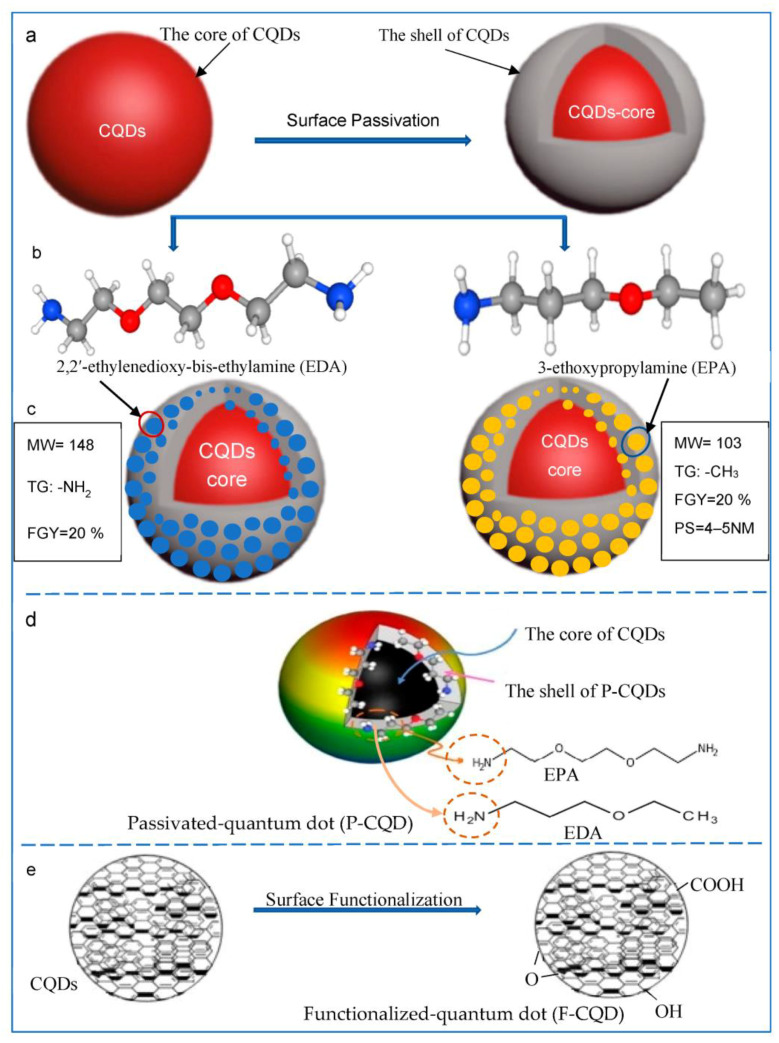
Schematic construction of surface modification of carbon quantum dots (CQDs): a surface passivation. (**a**) The spherical core and the thin layer shell of CQDs, (**b**) chemical structure of 2,2′-ethylenedioxy-bis-ethylamine (EDA) and 3-thoxypropylamine (EPA) which will be grafted on the CQD surface, (**c**) passivated CQDs, where MW is molecular weight of the surface molecule, TG is the terminal group of the surface molecule, FGY is fluorescence quantum yield, and PS is particle size. (**d**) 3D-ilustration schematic model for the grafted EDA and EPA, and (**e**) surface functionalization.

The surface modification of CQDs is an important target for selective application such as bioimaging and can be performed by either passivation (Figure 2a–d) or functionalization (Figure 2e) processes. The passivation process is the infliction of an outer layer of a shield material over a core material via a chemical reaction. This process is performed by constructing a core-shell model combined from passivation agents (such as EDA and EPA) that surround the hard fluorescent core of the CQDs and improve fluorescence emissions [31]. The process of surface functionalization (Figure 2e) involves adding functional groups to the surface, such as carboxyl, carbonyl, and amine groups, which can act as surface energy traps and change the fluorescence emission of CQDs. Surface chemistry or interactions such as coordination, interactions, covalent bonding, etc., can result in surface functionalization. The oxygenous characteristic of carbon quantum dots makes covalent bonding with functionalizing chemicals possible.

Functionalized carbon quantum dots have superior photoreversibility, high stability, strong biocompatibility, and minimal toxicity when compared to naked carbon quantum dots. Occasionally, a small number of molecules can serve as both passivating and functionalizing agents, requiring no extra post-synthesis modifications [29,32,33]. To compete with their rivals, such as organic dye molecules and inorganic semiconductor quantum dots, carbon quantum dots must have a high emission quantum yield. In addition to surface passivation and functionalization, one can use the heteroatom and nitrogen doping of carbon quantum dots to increase the quantum yield by up to 83%.

### 1.4. Applications of CQDs

#### 1.4.1. Industrial Field

CQDs have numerous applications in industrial fields [34] due to their enormous surface area, high electric conductivity, and quick electric charge transfer, as well as high physiochemical properties including crystallization, dispersibility in different liquids, and photoluminescence. In particular, the small size, superconductivity, and rapid electron transfer of CQDs endow the CQDs-based composites with improved electric conductivity and catalytic activity. In addition, CQDs have huge surficial functional groups that could facilitate the preparation of electrical active catalysts, which plays an important role in electrochemistry due to promoting charge transfer within and/or between molecules of these composites. By adjusting the size, shape, surface functional groups, and heteroatom doping of CQDs, it is possible to tailor their distinctive electrical and chemical structures. Rich organic groups that have been grafted onto the surface of CQDs make it possible for water molecules to easily adsorb there while also providing active coordinating sites for metal ions to produce CQD hybridized catalysts. The engineering of the electronic structures of the nearby carbon atoms within CQDs is greatly aided by the heteroatoms (such as N, S, and P) doped in CQDs [35].

Moreover, CQDs have been utilized to fabricate thin-film composite membranes for forward osmosis derived from oil palm biomass into polysulfone, which increased water flux and improved antibacterial performance [36] and nanofiller [37], packaging sheets [38,39], and lubricant additives [40].

Furthermore, there are many applications of CQDs in the field of electrocatalysis such as the reduction and/or evolution of oxygen, hydrogen, or CO_2_, as well as bifunctional catalysts, drug delivery, bioimaging, biosensing, optronic, solar cells, light-emitting diodes (LEDs), and fingerprint recovery [35].

#### 1.4.2. Medicinal Field

**Figure 3:**
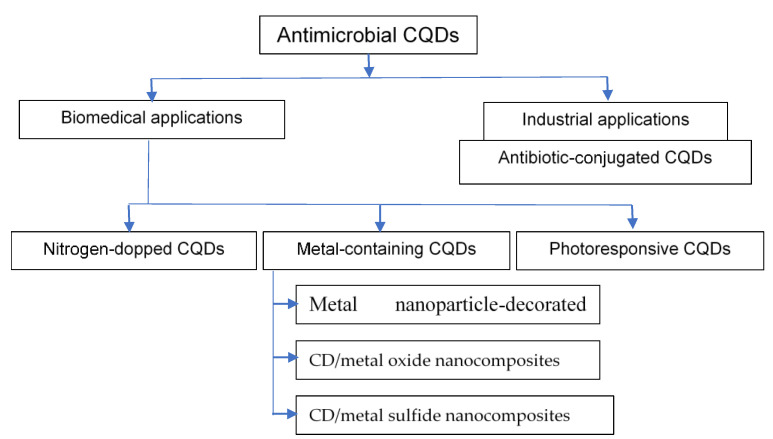
Scheme illustrating the different types of antimicrobial CQDs for biomedical and industrial applications.

The CQDs were reported to have medicinal therapeutic effects [15,16,24,40,41,42,43,44,45,46,47,48,49,50,51,52,53,54,55]. It was indicated that all these biomass-derived CQDs contain the nitrogen element, which might be from the proteins, amino acids, and nucleic acids in the biomass [34]. Furthermore, metal-containing CQDs (Figure 3) are divided into four types that can be used as antimicrobial agents: metal ion-doped CQDs, metal nanoparticle-decorated CQDs, CD/metal oxide nanocomposites, and CQD/metal sulfide nanocomposites [34]. For photoresponsive CQD, photosensitive agents (photosensitizers) are sensitized by light in the presence of oxygen to generate ROS, such as free radicals and singlet oxygen [56,57]

##### Bacterial Field

Several mechanisms were proposed to illustrate the effects of CQDs on typical bacterial cells [38,41,58,59,60,61,62]. The antimicrobial CQDs have been leveraged for coating the surface of orthopedic implant materials [58].

Positively charged CQDs (p-CQDs) effectively combat multidrug resistant (MDR) bacteria and can prevent the formation of biofilms, whereas n-CQDs significantly enhanced bone regeneration [41].

Incorporating water-dispersible and photoluminescent CQDs into bacterial nanocellulose (BNC) film was found to have protective activities against microbes, oxidants, and ultraviolet, making it suitable for food packaging [38]. The behavior of this biocomposite can be revealed by the hydrogen bonding interaction between CQDs and the surficial carboxyl, hydroxyl, and carbonyl groups of BNC, leading to the formation of the CQD–BNC film.

Bacterial biofilm (BB) is a key issue in the medical industry. The BBs were found to be colonized and to damage a wide range of medical implants and devices [59].

In addition, biofilms have major efficacy in many industries including oil, gas, and water production [60] due to causing metal corrosion in engineered systems.

In the complex process of biofilm formation, microorganisms grow and attach to surfaces in an irreversible manner. They also secrete extracellular polymeric substances (EPS) that help the formation of an extracellular matrix (ECM) and alter the phenotype of the organisms in terms of growth rate and gene transcription [61].

Although numerous conventional antimicrobial treatments have been employed to stop the development of mature biofilms or to remove them, these agents frequently require high dosages and are toxic, which poses serious risks to ecological and environmental systems as well as public health. Recent research on the newly created CQDs has had a substantial impact on efforts aimed at both prevention and eradication [11].

There are three general mechanisms illustrating the effects of CQDs on bacterial cells, namely electrostatic interaction, the disruption of the cytoplasm in which the internalization and intercalation occur in the bacterial membrane of the cytoplasm as a result of the charge alteration on the cell surface, and photodynamic inactivation with reactive oxygen species (ROS) production and DNA damage [62].

##### Viral Field

The semiconductor quantum dots can be used in labeling enveloped viruses for single virus trafficking [63]. Due to the importance of human noroviruses (NoVs), this review was focused on novel technical therapy using CQDs. NoVs are known for acute gastroenteritis outbreaks [64,65,66]. Great considerations were directed towards chemical and physical disinfection methods of human pathogens, especially norovirus (NoV) known as virus-like particles (VLPs) GI.1 and GII.4 [67,68]. This is due to the fact that there are currently no licensed vaccines or therapeutics for the prevention or treatment of human noroviruses. Moreover, a lack of well-defined infection models for such viruses, either in vitro or in vivo, has limited the development of their countermeasures [69]. Finally, these viruses are known for their resistance against traditional sanitizers and disinfectants [70]. However, most of these methods have been used for antibacterial applications and have been extended to be antiviral agents.

In the last couple of years, the use of nanoparticles as an antiviral strategy has gained much attention [14,15], which includes, but is not limited to, silver nanoparticles [71], gold-copper core-shell [72], TiO_2_ coupled with the illumination of low-pressure UV light [73], and passivated-carbon quantum dots (P-CQDs) which should be pithily considered [74].

A group of viruses known as NoVs (family: Calicivirdae) is distinguished by their single-stranded RNA and lack of an envelope. They consist of six genogroups (GI-GIV), which can be further divided into various genetic genotypes based on the sequencing of their capsids [64]. Examples of these are GI, which has nine genotypes, and GII, which has 22 genotypes [64]. It is worth mentioning that human infection is caused by the genogroups GI, GII, and GIV [75].

Gastroenteritis is a common cause of morbidity and mortality among all ages of individuals, and it results from a large variety of bacteria, parasites, and viruses [66]. Serovar is a distinct variation that may occur within a species of bacteria, virus, or immune cells, which can be used for classifying them according to their cell surface antigens.

It was reported by Patel et al. [66] that developing protocols for direct serovar purposes will be an important area of studying NoVs due to these viruses having not yet been cultivated. Expressed VLPs from different NoV strains were found to be useful as immunogens to produce hyperimmune animal sera, and as antigens to assess serum antibody responses to infection. Identifying a cellular NoV receptor and researching potential host–cell interactions have both been performed using VLPs. Human histo-blood group antigens (HBGAs) have been shown to function as NoV infection receptors.

It is known that histo-blood group antigens (HBGAs) determine the host’s susceptibility to NoV infection. Protection from viral infection is provided by antibodies that prevent NoVs–HBGAs binding [76]. The NoVs engage in strain-specific infection interactions with HBGAs in intestinal tissues as receptors or attachment factors [77,78]. It is important to note that HBGAs are terminal assemblies of glycan chains that are complex and highly polymorphic carbohydrates. They mostly consist of the ABO, secretor, and Lewis groups. Moreover, HBGAs are widely distributed on the mucosal epithelia of the gastrointestinal tract, where they serve as anchors for NoVs to begin infection [79]. According to earlier research, intestinal bacteria that express HBGA or synthetic HBGAs may promote NoV infection in B cells [80].

## 2. Material and Methods

### 2.1. Synthesis of CQDs

**Figure 4:**
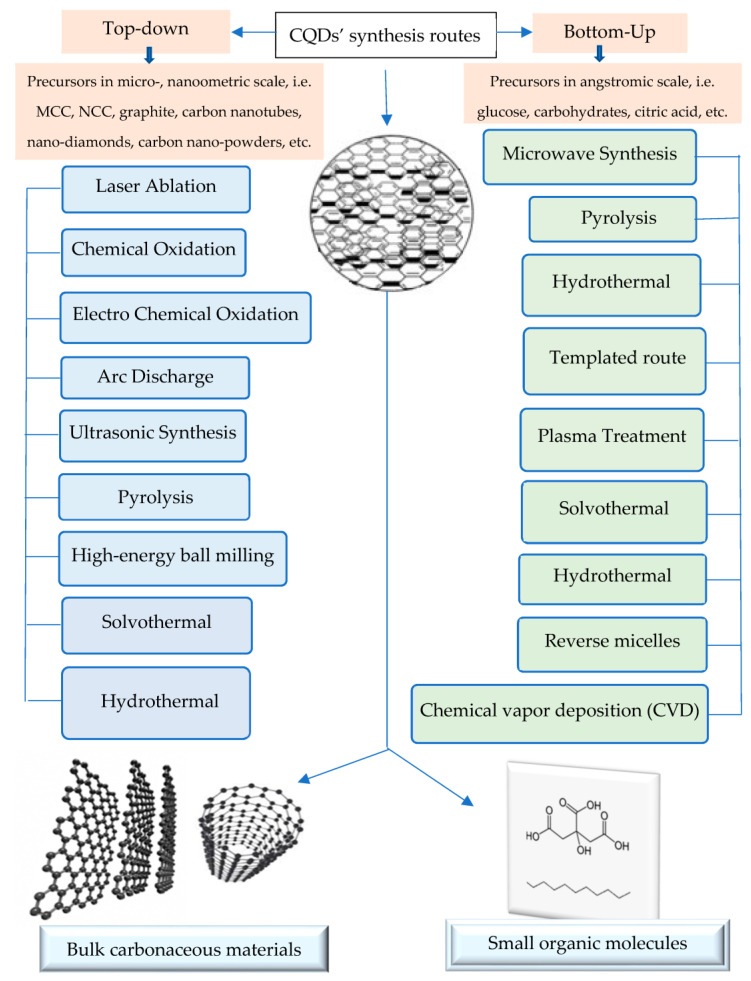
The synthesis routes of carbon quantum dots (CQDs).

For the synthesis of the CQDs, their precursor differs according to the synthesis route (Figure 4), either a top-down [25] or bottom-up route [81], and whether natural materials (Tables S1 and S2), especially nanocelluloses (MCC and NCC), are used, as shown in Table S2, or synthetic based precursors (Table S3). As shown in Figure 4, the ‘top-down’ synthetic route breaks down larger carbon assemblies such as graphite, carbon nanotubes, nano-diamonds, or carbon nano-powders [25] into CQDs below 10 nm. On the other hand, the ‘bottom-up’ synthetic route is a building process that begins from small precursors such as glucose, carbohydrates, citric acid, and polymer–silica nanocomposites [81].

The ‘top-down’ synthetic route breaks down larger carbon structures such as graphite, carbon nanotubes, nano-diamonds, or carbon nano-powders [25] into CQDs below 10 nm using laser ablation [59,74], arc discharge [82], high energy ball milling [83], and electrochemical techniques [84]. In addition, chemical oxidation with acid reinforces quick CODs with good characteristics [85,86,87].

On the other hand, the ‘bottom-up’ synthetic route is a building process that begins from small precursors such as glucose, carbohydrates, citric acid, and polymer–silica nanocomposites [81]. There are several synthesis methods via the bottom-up route, namely combustion/thermal/hydrothermal [88,89,90,91,92,93], plasma treatment [94], supported synthesis [95,96,97], solution chemistry approaches [91,92,98,99,100], and the cage-opening of fullerenes [101]. Regardless of their synthesis procedure, the resulting CQDs have different particle sizes, and thereby require complex separation processes to obtain mono-dispersed CQDs. Some of the explored post-synthesis separation techniques include dialysis [89], chromatography [84,102], gel electrophoresis [103], and ultra-filtration [104].

#### Synthesis of CQDs from Natural Resources

CQDs can be ingrained from plethora of macro-natural resources, as shown in Table S1 [11,16,105,106,107,108,109,110,111,112,113,114,115,116,117,118,119,120,121,122,123,124,125,126,127,128,129,130,131,132,133,134,135,136,137,138,139,140], as well as nano-natural resources of MCC and NCC (Tables S1 and S2) [3,6,7,8,9,141,142,143,144,145,146,147,148,149,150,151,152,153,154,155,156,157,158,159,160,161,162,163,164,165,166,167,168,169,170,171,172,173,174]. It was reported by Marpongahtun, et al. [11] that due to the fragmentation of the cellulose structure into tiny bits that carbonize to produce the CDs, CQDs were probably created during the thermal decomposition of the NCCs. Through a straightforward thermal pyrolysis method without any surface passivation, this work has successfully demonstrated the conversion of cellulose nanocrystals from oil palm empty fruit into fluorescing CQDs. The materials produced by pyrolysis at various temperatures exhibit various fluorescence and morphological characteristics.

Through a straightforward thermal pyrolysis method without any surface passivation, this work has successfully demonstrated the conversion of cellulose nanocrystals from oil palm empty fruit into fluorescing CQDs. The materials produced by pyrolysis at various temperatures exhibit various fluorescence and morphological characteristics.

##### Synthesis of Microcrystalline Cellulose (MCC)

The MCC can be synthesized by different processes such as reactive extrusion, enzyme mediated, steam explosion, and acid hydrolysis. The latter process is performed using mineral acids such as H_2_SO_4_, HCl, and HBr as well as ionic liquids (Table S2) in order to dissolve the amorphous regions, and, subsequently, the remaining the crystalline domains [6,7,8,9]. The degree of polymerization (DP) of the MCC is typically less than 400, while that for NCC is more than 400 extending to several thousands of (1→4)-β-d-glucopyranose units.

**Figure 5:**
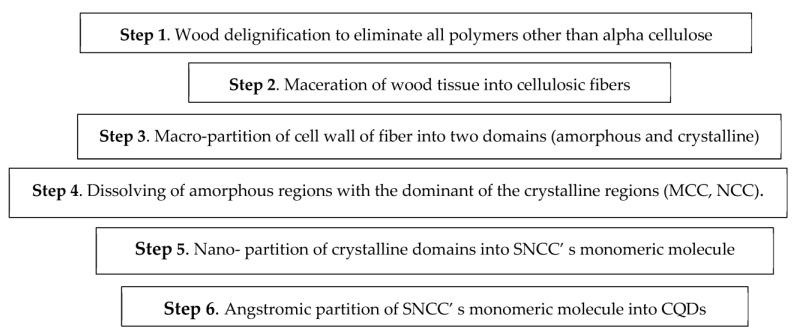
Schematic representation of deriving CQDs from macro-, nano-, and angstrom-structured cellulosic tissues.

**Figure 6:**
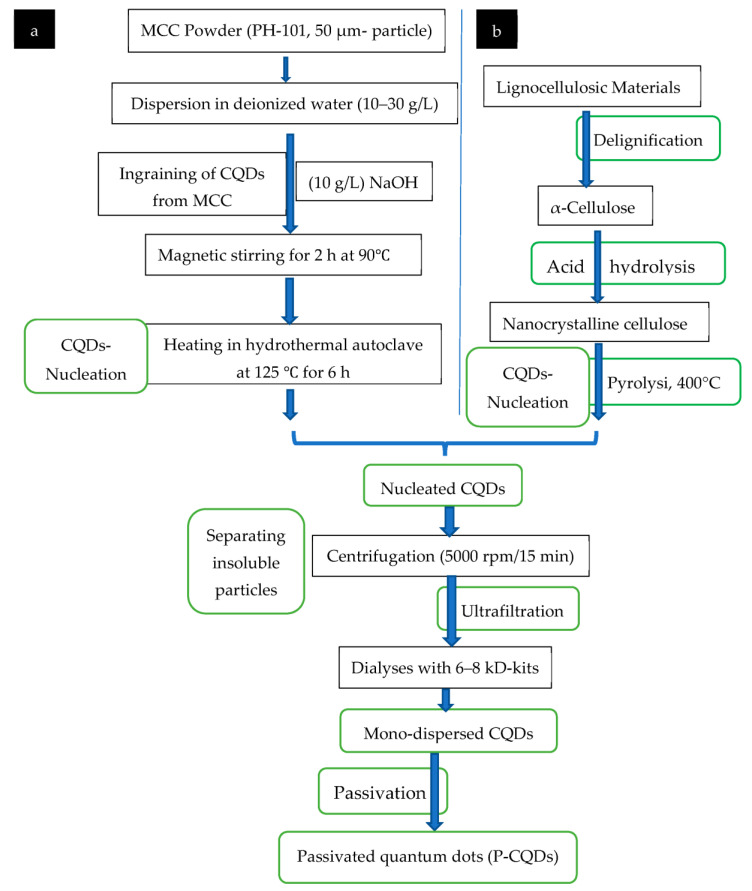
Synthesis of CQDs from (**a**) MCC, and (**b**) NCC [11,16].

After synthesizing MCC (Figure 5), CQDs were ingrained from MCC (Figure 6) and prepared under hydrothermal conditions [16].

##### Synthesis of Nanocrystalline Cellulose (NCC)

I. Ordinary Synthesis Methods

NCCs were synthesized by Hindi [3,5] by macerating cellulosic fibers with H_2_SO_4_, 64% *w*/*w* at 70 °C and stirring continuously for an hour. Deionized water was used to dilute the solution up to 20 times in order to stop the reaction. The unhydrolyzed fibers were removed from the suspension by centrifuging it at 1500 rpm, then for 20 min at 14,000 rpm to extract the NCCs. The precipitate was recovered, centrifuged again, and dialyzed until neutralized against deionized water. The NCC synthesis did not undergo any sonication exposure [3,5,6,7,8,9,10].

II. Cryogenic Synthesis Methods

A novel procedure for synthesizing NCCs, issued in December 2018, was invented by Hindi and Abohassan [6]. The patentability cornerstone of this patent is using liquid nitrogen vapors and/or its liquor for cooling the resultant NCCs to force them to be agglomerated, and, subsequently, precipitated. This cooling technique is termed as the lyophilizing process or cryogenic method. SEM and TEM analyses revealed that the obtained forced precipitates are nanoscale constructions (50–100 nm), although the agglomerated particles may reach up to several micrometers in diameter via the crystal growth phenomenon [6].

To obtain these NCCs, oven-dried MFs powder (10 g) is indirectly subjected to liquid nitrogen vapor. Then, once the frozen concentrated sulfuric acid (98.06%) is melted, it is allowed to saturate the lyophilized MFs powder in a ratio of 1:1 (wt/wt) by suction. A series of successive vacuums and releasing vacuums was performed as an alternative to the blending process to assist and accelerate the complete penetration of the acid into all interior pores of the MF structure. The acid-saturated MFs were re-lyophilized to maintain the synthesized crystalline particles from corrosion by the acid. Once the hydrolysis process had finished, a mixture of cold distilled water with tiny flakes in a ratio of 1:1 wt/wt was added to the NCCs synthesized. Then, two subsequent vacuum filtration steps were performed immediately after the dilution of the NCCs, namely primary filtration and secondary filtration. The primary filtration was performed using a textile (mesh), while the secondary filtration was applied using the Gooch crucible filter [6]. The simplicity of this patent is extended to cover the collection of the NCCs without needing to use the centrifugation process, obtaining nutrients’ NCCs via an ordinary washing process without needing to use the dialysis process, and using simple machinery helpful for cheap mass production of the NCCs.

III. Removal of NCCs’ sulfate groups

Sulfate groups were hydrolytically cleaved from CNCs following established procedures [12]. About 1% wt. dispersions of CNCs were treated with 1 M NaOH at 60 °C for 5 h. Then, the reaction was quenched by a 10-fold dilution with distilled water and centrifuged at 12,000 rpm at 4 °C for 20,121 min. Consequently, desulfated CNCs were re-dispersed and dialyzed against distilled water for one week to remove traces of NaOH.

##### Converting MCC into NCC

A simple, fast, economical, and ecofriendly method was invented for producing NCC from MCC using frozen concentrated H_2_SO_4_ and cooling with hair-shaped ice [10]. There are many benefits of using MCC as a starting material instead of cellulosic fibers for the synthesis of NCC, such as using less of the cellulosic precursor. As it consumes less concentrated acid, the MCC precursor can be easily handled within the synthesis apparatus because it is a powder, compared to the fibrous cellulose, and, finally, MCC is less susceptible to degradation by acid hydrolysis compared to other cellulosic precursors.

Other limitations of conventional NCC production processes include the requirement for the use of expensive machinery, such as sonication baths, sonication props, centrifuges, dryers, lyophilizers, and spray-driers or a complicated series of process steps such as requirements for centrifugation, sonication, neutralization, dialysis, and/or subsequent drying of an NCC product. Consequently, there is a need for a less complicated process that produces NCC in less time and at a lower cost.

After synthesizing NCCs, they are converted to CQDs in the manner illustrated in Figure 5 and Figure 6 [11,16].

### 2.2. Synthesis of CQDs from Synthetic Resources

**Figure 7:**
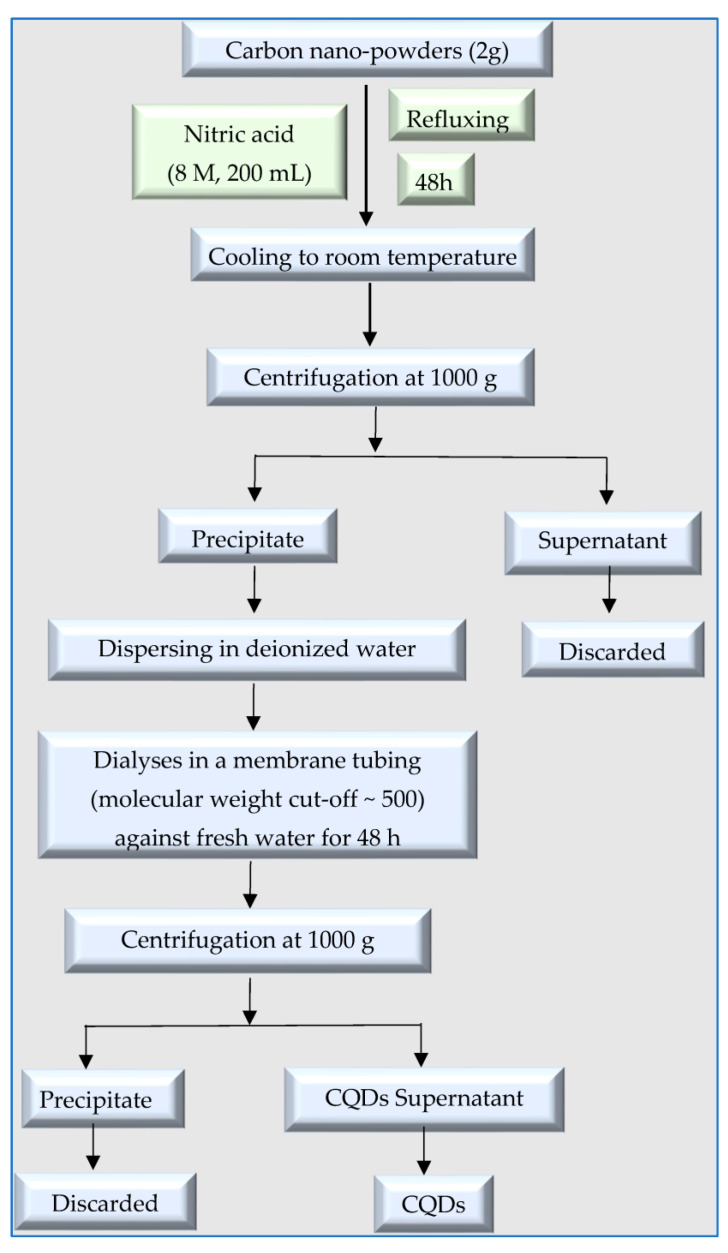
Synthesis of CQDs from carbon nanopowders.

Besides the possibility of synthesizing CQDs from natural resources, they can be produced from synthetic precursors such as suitable organic acids, salts, or carbonaceous materials, as presented in Table S3 [14,74,75,83,84,85,86,173,175,176,177,178,179,180,181,182,183,184,185,186,187,188,189,190,191,192]. Moreover, the CQDs can be synthesized from carbon nanopowders by the top-down route using nitric acid (8 M) under reflux for 48 h (Figure 7). After cooling the reaction liquor and centrifugation at 1000× *g*, the supernatant is discarded, while the precipitate is dispersed in water. The new liquor is dialyzed and centrifuged at 1000× *g* to retain the supernatant. Upon the subsequent dehydration, nanometric CQDs can be collected and are used in the subsequent functionalization process [15,29,32,33].

### 2.3. Syntheses of P-CQDs

The difference between the functionalization and passivation processes of a CQD to produce P-CQDs is shown in Figure 2. For the passivation process, two different P-CQDs can be synthesized, namely EDA-CQDs using 2,2′-ethylenedioxy-bis-ethylamine [15,29], and EPA-CQDs using 3-ethoxypropylamine, as presented in Figure 2b–d [15,29,32,33]. Furthermore, the surface functionalization of CQDs can be achieved by gifting chemical groups such as carboxyl, hydroxyl, oxygen atom, etc., to the CQDs’ surface (Figure 2e).

**Figure 8:**
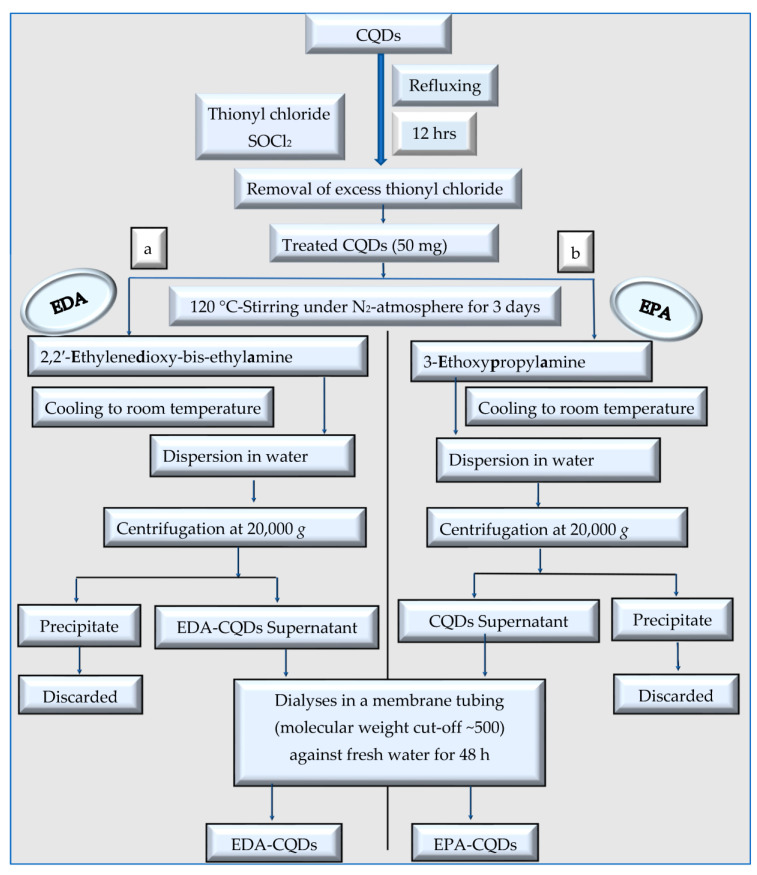
Synthesis of chemically passivated-CQDs (P-CQDs): (**a**) EDA-CQDs, and (**b**) EPA-CQDs [15,32,33].

As shown in Figure 2 and Figure 8, the synthesized CQDs are chemically passivated to yield either EDA- or EPA-CQDs [40]. First, the CQDs are allowed to react with SOCl_2_ in order to form the acid chloride intermediates which are more active than their carboxylic group precursors, and form amides by a reaction with the amine-terminated molecules [15].

#### 2.3.1. Characterization of Nanocelluloses

There are huge studies that were conducted to characterize the suitability of different natural precursors for the synthesis of CQDs [190,191].

In order to evaluate the MCC quality, several characteristics were tested, including particle size, density, compressibility index, angle of repose, powder porosity, hydration swelling capacity, moisture sorption capacity, moisture content, crystallinity index, crystallite size, and mechanical properties such as hardness and tensile strength. Furthermore, thermogravimetric analysis (TGA) and differential thermal analysis (DTA) or differential scanning calorimetry (DSC) are also important properties to evaluate the thermal behavior of the MCC under thermal stresses.

#### 2.3.2. Characterization of CQDs

Several techniques are used to characterize the CQDs such as nuclear magnetic resonance (NMR), X-ray diffraction (XRD), transmission electron microscope (TEM), Fourier transform infrared spectroscopy (FTIR), fluorescence spectrophotometer, ultraviolet (UV) spectroscopy, UV–vis absorption spectra, and atomic force microscopy (AFM), as reported by Xu et al. [192], Singh et al. [59,193], and Joo et al. [63]. Moreover, the interactions of various polypeptides with individual carbon nanotubes (CNTs), both multiwall (MW) and single wall (SW), were investigated by Li et al. [194] using atomic force microscopy (AFM). The characterization procedures were performed to test for bacteria [13,61,195,196,197] and viruses [15,16,24,197,198,199,200,201,202,203,204,205,206,207,208,209].

#### 2.3.3. Evaluation of Viral Therapeutic Efficacy of P-CQDs

Briefly, saliva samples from healthy adult volunteers, including blood type A, B, and O, are collected [15]. The pretreatment process is performed using phosphate-buffered saline (PBS). The plates are blocked with Super-Block T20 (PBS) blocking buffer as shown in Figure S5. Collected saliva samples from individuals with blood types A, B, and O are collected and pretreated with phosphate-buffered saline (PBS). The samples are immediately boiled for 5 min and centrifuged at 10,000× *g* for 5 min. The collected supernatant is diluted to 1:2000 in PBS. For coating the plates with HBGAs, an aliquot of 50 μL saliva dilution was used to coat 96-well plates at 4 °C overnight. Unbound saliva was removed and the wells were rinsed three times with super-block T20 (PBS) buffer to yield a high signal-to-noise ratio in the detection system [15]. Noticeably, a similar difference in effectiveness between EDA-CQDs and EPA-CQDs was found in their antiviral function [27], where EDA-CQDs were more effective than EPA-CQDs in inhibiting norovirus virus-like particles binding to histo-blood group antigen receptors, due primarily to the difference in surface charge status between the two CQDs.

The final solution is treated with 3,3′,5,5′-tetramethylbenzidine (TMB) peroxidase developer, and the absorbance is measured at the wavelength of 450 nm using a microplate reader (Figure S2).

As presented at Figure S3 [15,77,79], the enzyme-linked immunosorbent assay (ELISA) test is used to evaluate the binding capacity between the EDA-CQDs and EPA-CQDs and human NoVs–VLPs antibody (GI.1 or GII.4) using two standard antibodies: (1) primary antibody, namely mAb 3901 for the strain ‘GI.1’, or mAb NS14 for the strain GII.4, and (2) secondary antibody such as horseradish peroxidase (HRP) having 44,173.9-dalton glycoprotein with 6 lysine residues for labeling goat anti-mouse IgG antibody [15]. It produces a colored, fluorometric, or luminescent derivative of the labeled molecule when incubated with a proper substrate, allowing it to be detected and quantified [194].

The sodium dodecyl sulphate polyacrylamide gel electrophoresis (SDS-PAGE) test and Western blotting protocol (Figure S4) are used for evaluating the effect of EDA- and EPA-CQDs on VLP capsid protein. Different concentrations of EDA- and EPA-CQDs (20 or 60 μg/mL) are applied to treat VLPs (GI.1 or GII.4) [15]. 

On medium-binding 96-well polystyrene plates, EDA- and EPA-CQDs at various doses ranging from 0 to 60 g/mL are employed to treat either GI.1 or GII.4 VLPs. The reaction solutions are removed and the wells are twice washed with phosphate-buffered saline (PBS) following the addition of a specific amount of PBS, agitation, and incubation for 30 min.

For one hour, PBS-blocking buffer is used to block the wells. Each well is twice washed with phosphate buffered saline with tween 20 (PBST) buffer after the blocking solution has been discarded. After that, 50 aliquots of 1 g/mL anti-GI are added. To interact with the bound GI.1 or GII.4 VLPs, 1 VLP antibody (mAb 3901) or anti-GII.4 VLP antibody (mAb NS14) is added to each well. Each well is put into a solution containing goat anti-mouse IgG that has been HRP-labeled before being incubated at 37 °C for 1 h. The wells are then twice rinsed with PBST. PBST is used to wash the plates. Tetramethylbenzidine (TMB) peroxidase is used to create the end product, and its absorbance is measured at 450 nm.

The gel containing the VLPs-treated P-CQDs (GI.1-VLP/EDA-CQDs and GI.1-VLP/EPA-CQDs) is used for staining (Figure S5) and Western blotting (Figure S6). The gel used for staining was previously prefixed with a 50% methanol and 7% acetic acid solution, stained by GelCode Blue stain, and imaged using an infrared imaging system as explained.

For 30 min, 1.5 mL centrifuge tubes were continuously shaken at the setting level of 2 at an ambient temperature. Following the CQDs treatments, 5 μL of 1 × NuPAGE LDS sample buffer, 2 μL of 1 M DTT, and 3 μL deionized water were added to each tube. After 10 min of incubation at 70 to 80 °C, all of the samples were placed onto 2 precast 1.0 mm × 10-well NuPAGE^®^ 4–12% Bis-Tris gels (Life Technologies, Grand Island, NY, USA). For each well, the loading volume was adjusted to be 10 L.

The gels were run for one hour at 200 V in 1 MOPS SDS running buffer. One gel was used for Western blotting, and the other was used for staining. The gel for staining was pretreated for 15 min with a solution of 50% methanol and 7% acetic acid, and then washed three times for 5 min with deionized water. The GelCode Blue stain was applied to the gel and shaken continuously for 1 h before being washed with deionized water for 1 h to remove the stain. Infrared imaging equipment was then used to image the gel (Figure S5).

As presented at Figure S6, regarding Western blotting, the gel is treated with NuPAGE^®^ Transfer Buffer and 10% MeOH packaged within the nitrocellulose membrane using Hoefer Semi-Dry Transfer Apparatus. The membrane is blocked with blocking buffer and PBS.

The gel was transferred to a nitrocellulose membrane for Western blotting (Figure S6), which is blocked using blocking buffer and PBS at room temperature for one hour. Both GI.1/antibody mAb 3901 and GII.4/antibody mAb NS14 underwent primary antibody treatment using PBST and blocking buffer. After incubating the antibody solution at 4 °C with gentle shaking for the entire night, it was discarded. The membrane was treated with 0.5 μg of goat anti-mouse IRDye^®^ 800CW antibodies in PBST and blocking buffer at ambient temperature for 1 h after being rinsed 5 times with PBS plus 0.05% Tween 20 (PBST) for 5 min each time. The membrane was first washed with PBST five times for approximately five minutes each while being shaken, followed by a soak in deionized water, and then an IR imaging system was applied (Figure S6).

## 3. Results and Discussion

The properties of ordinary CQDs as well as P-CQDs (EDA- and EPA-CQDs) and their inhibitory rate on microbial defense are presented in Table 1 (for viruses) and Table 2 (for bacteria and fungi).

### 3.1. Viral Therapy of P-CQDs on NoVs

#### 3.1.1. Absolute Efficacy

P-CQDs have an inhibitory effect on the binding of VLPs to HBGA receptors. Human HBGAs are recognized by NoVs as attachment factors or receptors having a significant impact on the host’s susceptibility to NoV infection [200,201]. It has been discovered that norovirus binding to HBGAs is extremely varied but strain-specific.

Based on the binding of different norovirus strains to HBGAs, several binding patterns have been discovered and divided into two primary binding groups [206], and a model of norovirus/HBGA binding has also been put forth [78]. Other studies revealed that Norwalk VLPs lacked the binding to saliva samples obtained from nonsecretors, and that saliva from type B individuals did not bind or only weakly bound to the Norwalk virus [199]. A retrospective study revealed that type O individuals had a significantly higher infection rate than those with other blood types [198].

The approximate mean values obtained from studying the impact of EDA- and EPA-CQDs on the binding of GI.1 and GII.4 VLPs to salivary HBGAs from blood type A, B, and O are reported in Table 1. The binding to type ‘A’ salivary HBGA receptors was entirely blocked for GI.1 VLPs treated with EDA-CQDs at 5 g/mL (100% inhibition), demonstrating a highly effective inhibition impact of EDA-CQDs on GI.1 VLP’s binding to HBGA receptors.

When GII.4 VLP bound to type A HBGA receptors was treated with 5 g/mL EDA-CQDs, the same quantitative inhibition (100%) was seen (Table 1). Even at lower CQD concentrations, the inhibitory effect persisted, as seen by the more than 80% inhibition in GI.1 and GII.4 VLP bindings after treatment with 2 g/mL EDA-CQDs (Table 1).

The findings revealed that the diverse strains of VLPs had a similar inhibitory effect to EDA-CQDs on HBGA receptor binding. Although slightly less potent on a rising concentration basis, EPA-CQDs were still quite effective in the same inhibition.

As seen in Table 1, treatment with EPA-CQDs at concentrations of 5 g/mL and 2 g/mL inhibited the binding of GI.1 VLPs to type A HBGA receptors by 91% and 51%, respectively. A similar suppression of GII.4 VLPs was seen after treatment with EPA-CQDs (Table 1). These results demonstrate that EDA- and EPA-CQDs had equally potent inhibitory effects on the two strains of VLPs’ ability to bind to type B and type O HBGA receptors. Investigating the inhibition to type A HBGA receptors, shown in Table 1, also exhibited the dot concentration dependence and difference between the two types of CQDs (EDA- and EPA-CQDs). The findings for the two different strains of VLPs indicated that EDA-CQDs were more efficient than those for EPA-CQDs in preventing VLP binding to all three types of HBGA receptors.

The differing surface charge status and hydrophobicity characteristics between the two types of CQDs may be to blame for the different effectiveness. While EPA-CQDs with surface methyl (-CH_3_) terminal groups are not charged, EDA-CQDs with surface amino (-NH_2_) terminal groups tend to be altered positively at physiological pH (-NH_3_^+^).

Even though the mechanistic details of the interactions of the CQDs with the VLPs and the resulting inhibition effects are probably very complex [78,80], the negatively charged VLPs should be more attractive to the positively charged EDA-CQDs, leading to a higher “local concentration” of the dots around the VLP particles. One of the potential explanations for the CQDs’ observed strong inhibitory effects is that they bind to the surface of the VLPs and physically block the binding sites for the HBGA receptors.

According to the X-ray crystal structure of the NoVs prototype GI.1 [207], it has two domains, namely the shell (S) domain and the protrusion (P) domain. The HBGA receptor binding interfaces are found at the top of the ‘P’ domain and contain pockets for binding carbohydrates. The binding of HBGAs to the viral capsid protein is stabilized by these pockets, which include many dispersed amino acid residues that form large hydrogen bond networks with individual saccharides [208,209]. However, some of the complexities in the HBGA binding interactions have been reported [11], including capsid P domain loop movements, alternative HBGA conformations, and HBGA rotations. This is because the binding of norovirus to human HBGA is a typical protein–carbohydrate interaction in which the protruding domain of the viral capsid protein serves as an interface for the oligosaccharide side-chains of the HBGAs40.

In fact, employing sera from immunized animals or sick humans aids the blockage of NoV HBGA binding sites and has been employed as a proxy for a NoV neutralization experiment [202,203]. It was discovered that protection against infection in NoV-vaccinated chimpanzees and against sickness in infected human volunteers could be connected with the serum’s capacity to prevent VLP–HBGA interactions [65,76]. These investigations suggest that a promising method for avoiding HuNoV infection is to inhibit the HuNoV capsid from recognizing its binding sites on host cells. As a result, the CQDs reported efficient inhibition of the NoV VLPs, as shown in Table 1; it may be viewed as an application of this tactic.

**Figure 9:**
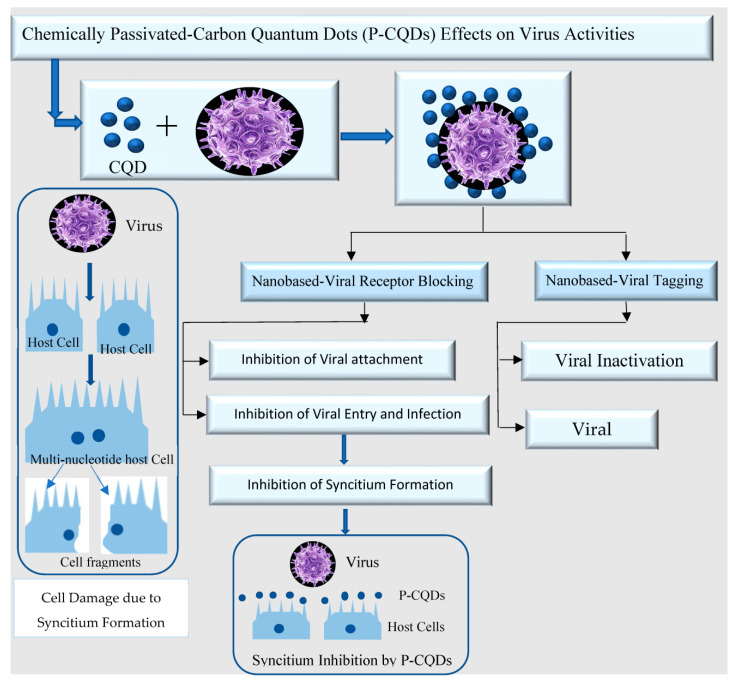
The potential role of the passivated-carbon quantum dots (P-CQDs) in detecting and inactivating human infection by noroviruses through targeted tagging and by blocking viral surface proteins.

In addition, the antiviral activities of the P-CQDs are summarized in Figure 9. NoV infects the host cell via surficial cell receptors leading to the formation of syncytium and subsequent gradual degradation, which are responsible for spreading viral infection. With the recent advances in nanotechnology, NoV infection can be detected and inactivated specifically through different pathways such as targeted tagging or by blocking surficial viral proteins.

It is well known that HBGAs, as receptors, play an essential role for host susceptibility to NoV infection [189,210]. Although the binding between NoVs and HBGAs is highly diverse, it is strain-specific. Numerous patterns of such binding have been identified and classified into two major groups, proposing a suitable model [73]. It was indicated by Hutson et al. [198] that type O individuals had a significantly higher infection susceptibility rate than those with other blood types. On the other hand, other studies showed that Norwalk VLPs did not bind to saliva samples collected from nonsecretors, especially for type B individuals [198].

Both EDA- and EPA-CQDs showed a strong inhibition effect in the binding between the two strains of VLPs and the HBGA samples collected from type B- and type O-individual receptors [15]. Furthermore, the EDA-CQDs were found to be more effective than EPA-CQDs in inhibiting VLPs’ binding to HBGA receptors. This difference may be attributed to the quality (positive, negative, or neutral) and amount of surficial charge for the P-CQDs as well as the virus surface. EDA-CQDs with the surficial terminal amino (-NH_2_) groups are positively charged at physiological pH (-NH^3+^), whereas EPA-CQDs with surficial terminal methyl (-CH_3_) groups are not charged. As VLPs are negatively charged, they are perhaps more attractive to the positively charged EDA-CQDs, leading to a higher accumulation of the P-CQDs around the NoV particles. However, the mechanism of the interactions between the P-CQDs and viruses is likely very complex [78,80].

The strong inhibitory effects of P-CQDs against the NoVs can be attributed to the physical blocking that occurred as a result of the binding between the P-CQDs and the surficial active sites on the virus. XRD investigations revealed that the NoV strain ‘GI.1’ contains two domains: (1) the shell domain (SD), and (2) the protruding domain (PD) which contains the HBGA–carbohydrate complex formed via a hydrogen bond network [198]. Accordingly, the binding between the human HBGA and P-domain is a carbohydrate–protein complex [211]. Some of these complexes include movements of the binding interaction as well as conformations and/or rotations of the HBGA. The blocking of NoVs’ binding sites by using sera from immunized individuals could be classified under such a strategy [80,201,202]. Moreover, Dong et al. [15] showed that the EDA-CQDs are more effective in the inhibition of the NoVs (GI.1), binding to the first antibody (mAb 3901) compared to binding to the GII.4-mAb NS14. This difference between EDA- and EPA-CQDs may be attributed to their difference in surficial charge status.

In addition, for both EDA- and EPA-CQDs treatments using different concentrations, the NoV-strain ‘GI.1’ was inhibited in its binding to mAb3901 antibodies more effectively than the strain ‘GII.4’ in its binding to mAb NS14 [15]. This might be due to the capsid structure difference in the two strains of NoVs (GI.1 and GII.4), involving in NoVs–antibody interactions. Furthermore, no significant difference was detected in the P-CQDs inhibitory effect on the binding of both strains of NoVs to HBGA receptors [15]. 

After the P-CQDs treatments, the quantity of NoVs fragments found by Western blotting was found to be unchanged. It is known that mAb 3901-antibody may bind to both the full-lengths of 58 KDa and a 32 KDa of the protein fragments found in the P domain of the NoVs strain (GI.I) protein bands [203,204,205], identifying a continuous epitope on the C-terminal of the capsid protein [205]. Furthermore, the lowest band in the Western blot is probably a fragment that contains this sequence because the antibody mAb 3901 also identifies a domain between amino acids 453 and 495. The mAb NS 14-antibody binds to the capsid protein and additional protein fragments that contain the identified epitopes, just like it does for the other NoVs strain (GII.4). Obviously, the protein band patterns found in Western blotting for both strains of the NoVs (GI.1 and GII.4) were nearly identical to those found in SDS-PAGE found by GelCode Blue staining. Therefore, the findings portray that the viral proteins were not degraded by the P-CQDs, since these proteins still retained their virgin sequences of the amino acids and were able to react with their antibodies again [15].

#### 3.1.2. Comparative Efficacy for Other Carbon Nanomaterials (CNMs)

The most crucial characteristics governing the behavior of CQDs and subsequent applications are absorption, photoluminescence (PL), and electroluminescence [28]. Generally, the optical absorption peaks of CQDs in the UV-visible region are usually estimated as the π-π* transition of sp^2^ conjugated carbon and n-π* transition of hybridization with a heteroatom such as N, S, P, etc. Surface passivation or modification processes can be used to modify the absorption property [35].

The PL is one of the most wonderful features of CQDs. Generally, the distinct dependence of the emission wavelength and intensity is one of the uniform features of the PL for CQDs. The reason for this unique phenomenon may be the optical selection of nanoparticles with a different size, or CQDs with different emissive traps on the surface. The variation in particle size and PL emission can be reflected from the broad and excitation-dependent PL emission spectrum [73,74].

Zhang et al. [79] studied the emission behaviors of CQDs at 470 nm wavelength with various concentrations. It was found that the PL strength of the CQDs solution first increased and then decreased with the increase in their concentration [35].

Similar to semiconductor nanocrystals, CQDs can display electroluminescence (ECL), which can be used in electrochemical fields [35]. It was reported by Zhang et al. [79] that a CQDs-based light-emitting diodes (LED) device could be used, in which the emission color ranging from blue to white can be controlled by the driving current.

In order to comprehend the luminescence process of CQDs based on the band gap emission of the conjugated p domain and the edge effect generated by another surface defect, two models of CQDs were put forth by SK et al. [19]. The quantum confinement effect (QCE) of p-conjugated electrons in the sp^2^ atomic framework is the source of the photoluminescence (PL) features of the fluorescence emission of CQDs from the conjugated p domain, which may be modified by altering their size, edge configuration, and shape. The sp^2^ and sp^3^ hybridized carbon and other surface defects of CQDs cause fluorescence emission, and even the fluorescence intensity and peak position are connected to this defect.

At low pHs, the interaction is dominated by adhesion forces resulting from electrostatic interactions between the protonated amine groups of polylysine and carboxylic groups on acid-oxidized multi-wall carbon nanotubes (Ox-MWCNTs), whereas at high pHs, adhesion forces via hydrogen bonding between the neutral -NH_2_ groups of polylysine and the -COO^−^ groups of the Ox-MWCNTs are detected [193].

Furthermore, it was discovered that the adhesion force for oxidized multiwalled carbon nanotubes (Ox-MWCNTs) increased with the oxidation time, while it was negligible for oxidized single-wall carbon nanotubes (Ox-SWCNTs) because the latter had carboxylate groups attached only to the nanotube tips as opposed to both the sidewall and the tips. Additionally, it was shown that proteins with aromatic moieties, such as poly-tryptophan, exhibited a stronger adhesion force with Ox-MWCNTs than polylysine because of the additional pi-pi stacking interaction between the polytryptophan chains and CNTs. [193].

The binding ability between various CNMs and viral capsid proteins has been reported [17,18,20,21,193]. The CNTs and P-CQDs can be non-specific binders to NoVs’ capsid proteins through complementary charges, π-π stacking, and/or hydrophobic interactions [17,18,194].

It was reported that van der Waals forces are responsible for the binding between fullerene (C_60_) and lysozyme, whereas polar solvation and entropy were reported to be detrimental to this binding [20]. Furthermore, C_60_ was reported to inactivate HIV-proteases by integrating with proteins to form hybrid functional assemblies [21]. Similarly, the inhibition of NoVs’ capsid protein by the P-CQDs may occur due to the combination of several driving forces for blocking the active sites on NoVs with the HBGA receptors [15].

The van der Waals force was found to be the primary driving factor responsible for the binding between fullerene and lysozyme, whereas polar solvation and entropy are detrimental to such bindings [20]. It was shown that C60 might suppress the activity of HIV proteases by integrating with proteins to form hybrid functional assemblies [21], which is more pertinent to the blockage of receptor sites.

As a result, a conceptually similar explanation for the observed inhibition of NoVs-VLPs could be that the CQDs interact with the capsid protein of VLPs by combining several driving forces, which then prevents the active sites on NoVs-VLPs from binding to HBGA receptors.

In addition, a similar surface charge effect has been reported on silver nanoparticles’ antimicrobial activity, where positively and negatively charged silver nanoparticles exhibited the highest and lowest bactericidal activities, respectively [29]. As such, there have been recent studies on inducing charges onto the surface of silver nanoparticles for higher antimicrobial efficacy [32,33,200]. The results reported here suggest that the same strategy may be exploited in the design and preparation of CQDs with higher antibacterial efficacy [15].

### 3.2. Bacterial Therapy Efficacy of P-CQDs

Biofilm formation is a complex process in which microorganisms irreversibly attach to and grow on a surface and produce extracellular polymeric substances (EPS) that facilitate the attachment and formation of an extracellular matrix (ECM), resulting in the altered phenotype of the organisms with respect to growth rate and gene transcription [61].

Important characteristics of EDA-CQDs and EPA-CQDs are listed in Table 2. A Gram-positive laboratory model bacteria, *Bacillus subtilis*, was used to evaluate the antimicrobial efficiencies of each of the CQDs with different surface passivation (EDA-CQDs and EPA-CQDs) for probing the surface charge effect. As is clear in Table 2, EDA- and EPA are small molecules, with molecular weights of 148 and 103 g/mol, respectively, and they are structurally similar but their corresponding CQDs differ in terms of terminal groups on the dot surface, -NH_2_ in EDA-CQDs vs. -CH_3_ in EPA-CQDs. The former can be positively charged at physiological pH as -NH_3_^+^, but not the latter. The observed fluorescent quantum yields of the EDA-CQDs and EPA-CQDs used in the study were both ~20% [196].

Additionally, it is evident from Table 2 that EPA-CQDs and EDA-CQDs at 0.1 and 0.2 mg/mL to *Bacillus subtilis* cells have antibacterial action in terms of a reduction in viable cell counts after treatments with light illumination for one hour. At 0.1 mg/mL, EPA-CQD treatment minimally reduced the number of viable *Bacillus subtilis* cells, but EDA-CQD therapy was significantly more successful, causing a 3.26 log drop in viable cells.

EPA-CQD treatment reduced the number of viable *Bacillus subtilis* cells by about 0.84 log at a CQD concentration of 0.2 mg/mL, whereas EDA-CQD treatment reduced the number of viable cells by around 5.8 log at the same concentration. EDA-CQDs consistently outperformed EPA-CQDs in terms of their antibacterial action toward *Bacillus subtilis* cells at both tested CQD doses, as was to be expected.

These findings demonstrated how crucial surface charge is for CQD interactions with bacteria and the performance of their antibacterial activity. Stronger binding-like interactions between EDA-CQDs and the bacterial cells will result in a higher “local concentration” of EDA-CQDs on the bacterial surface, making them more effective in antibacterial actions against the bacterial cells [196]. The negatively charged bacterial surface must favor the positively charged end groups (-NH_3_^+^) on EDA.

As is clear from Table 2, using 10 μg/mL of the EDA-CQDs is very effective in inhibiting the biofilm formation for all the addition times used (1, 2 and 3 h) compared to 20 and 30 μg/mL, as is also indicated by Dong et al. [13]. When the 10 μg/mL CQDs were added at 1, 2, and 3 h after the initiation of biofilm growth, the inhibitory effect on the final biofilm formation was decreased from 95.86% (at 1 h) to 72.2% (at 2 h) reaching to about 34.25% (3 h), as indicated by different researchers [13,61,197]. Furthermore, the time of CQDs’ addition during biofilm growth had a significant effect on the process of biofilm formation up to the final product stage.

These results are logical when considering the interactions between EDA-CQDs and bacterial cells during biofilm formation. At the early stage during biofilm formation, no thick extracellular polymeric substances (EPS) are produced around the bacteria, and most of the bacterial cells are still planktonic so that the added EDA-CQDs can bind and interact with bacteria efficiently to inactivate the cells before they can form a biofilm; thus, this explains the observed high inhibitory effects on biofilm formation.

In addition, these findings demonstrated how crucial surface charge is for CQD interactions with bacteria and the performance of their antibacterial activity. Stronger binding-like interactions between EDA-CQDs and the bacterial cells will result in a higher “local concentration” of EDA-CQDs on the bacterial surface, making them more effective in antibacterial actions against the bacterial cells [196]. The negatively charged bacterial surface must favor the positively charged end groups (-NH_3_^+^) on EDA.

Bacterial cells multiply and the extracellular matrix (ECM) gradually becomes stronger with the development of biofilm if CQDs are added 4–5 h after the start of biofilm growth. As the bacteria expand, the development of an ECM network may make it more difficult for CQDs to enter the biofilm and for EDA-CQDs to interact directly with the bacterial cells. These contacts and interactions are especially important to the light-activated EDA-CQDs’ antibacterial function.

The production of electrons and holes, which are trapped at various stabilized surface defect sites, requires quick charge transfers and separation for a better representation of photoexcitation of the EDA-CQDs. Due to the short half-lives of these redox species, these separated redox pairs are attributed with making significant contributions to the observed antibacterial activities [13,197], largely in the near-neighbor manner due to the short-lived nature of these redox species. Their radiative recombinations produce emissive excited states that are responsible for the fluorescence’s noticeable brightness and color, as well as the production of traditional reactive oxygen species (ROS), which also aid in the antibacterial effect. Although the ROS are still transient, the poor diffusion circumstances caused by the ECM network during the biofilm formation may also interfere with their antibacterial properties.

Due to the restriction associated with the requirement for the CQDs to penetrate into the biofilm, EDA-CQDs with light activation are therefore more effective in preventing biofilm formation before the bacterial cells have the chance and time to form the network structure toward the biofilm, and are less effective when the biofilm formation is already well underway. This restriction was made clearer in an investigation of the removal of mature biofilms using EDA-CQDs and the same visible light exposure [13].

Moreover, based on the investigation performed by Mogharbel et al. [16] who examined the microbicide potency for the embedded CQDs against three distinct bacterial strains, including a Gram-positive bacterial strain (*Staph. aureus*), Gram-negative bacterial strain (*Escherichia coli*), and fungal strain (*C. albicans*), as shown in Table 2, the superior microbicide potency of CQDs against several bacterial strains has been confirmed [13,16,61,197]. This effect was attributed to the decorative hydroxyl groups: (i) decorative oxygen-containing groups are responsible for the mortal effects of the prepared CQDs against the tested microbial cells through the generation of reactive oxygen species (ROS); (ii) the liberated ROS act by killing the microbial cells, as ROS adhere to them and then penetrate the microbial cell wall to motivate the oxidative stress by deteriorating DNA and RNA.

Additionally, ROS contribute to mitochondrial dysfunction, lipid peroxidation, inhibition of intracellular protein synthesis, progressive deterioration of the cell wall, and, ultimately, apoptotic cell death. The efficiency of hydrothermal conditions in the formation of small and size-controllable CQDs that are easily able to penetrate the microbial cell wall for eventual cell demise is attributed to the fact that CQDs-HT demonstrated significantly higher microbicide potentiality [16].

## 4. Conclusions and Future Perspectives

Biomass has a carbon chain which is why it is considered as an excellent option for the production of carbon materials. Nanocrystalline cellulose could become a potential source for fabricating carbon quantum dots which are affected by pyrolysis temperature.

The large surface area, good electric conductivity, and fast electric charge transfer of carbon quantum dots endow them with a great potential for a wide spectrum of applications. Luminescent carbon quantum dots are interesting newcomers in the category of nanomaterials, emerging with more and more advanced applications in the fields of chemical sensors, bioimaging, nanomedicine, drug delivery, and electrocatalysis. The unique electronic and chemical structures of carbon quantum dots can be tuned by controlling their size, shape, surficial functional groups, and heteroatom doping.

The 2,2′-ethylenedioxy-bis-ethylamine-carbon quantum dot and 3-ethoxypropylamine-carbon quantum dot were found to be highly effective to inhibit noroviruses from binding to histo-blood group antigens receptors on human cells with inhibition efficiencies of 100% and 85–99%, respectively.

In the future, we hope to discover more precursors and invent more economic, simple, and innovative synthetic methods and novel promising applications to increase the potential of these valuable carbon materials. In addition, more efforts must be made to simplify the traditional machinery used for the synthesis process of carbon quantum dots, especially in the collection of the nanometric dots by centrifugation, the neutralization of the dot’s supernatant by dialysis, and the standardization of the dot size. Furthermore, the good findings in regard to using chemically-passivated carbon quantum dots for the prevention and therapy of norovirus must be extended to cover other epidemic pathogens, especially coronaviruses (COVID-19).

## Figures and Tables

**Table 1:** Estimated mean values ^1^ of the P-CQDs for inhibition of the NoVs.

Property of the CQDs	^2^ HBGA’s Type	Concentration µg.mL^−1^	VLS Strains	P-CQDs	
				EDA	EPA
Inhibitory rate of CQDs on NoVs, %		8		90	8
		16		92	24
		32		88	26
Inhibition of HBGA binding, %	A	2	GI.1	93.6	53.3
			GII.2	88	61.2
		5	GI.1	100	93
			GII.2	100	100
	B	2	GI.1	74.5	36.4
			GII.2	78.2	38.2
		5	GI.1	99.1	75.5
			GII.2	100	81.8
	O	2	GI.1	79.1	54.4
			GII.2	59.5	38.9
		5	GI.1	100	77.2
			GII.2	100	79.3

^1^ Mathematically-estimated from Dong et al. [15]; ^2^ Histo-blood group antigens.

**Table 2:** Mean values of the P-CQDs for inhibition of bacteria.

Property of the CQDs		^1^ AT Hour	^2^ Conc. µg.mL^−1^	P-CQDs		CQDs		Reference
				EDA	EPA	^3^ HT	^4^ IR	
Particle size, nm				4–5	4–5			[200]
Molecular weight				148	103			
Surficial terminal group				-NH_2_	-CH_3_			
Fluorescence quantum yield, %				~20	~20			
Viable cell number of bacteria, CFU/mL			0	11 × 10^6^	11 × 10^6^			
			0.1	9 × 10^3^	5 × 10^6^			
			0.2	0.4 × 10^3^	1.5 × 10^6^			
Inhibitory effect OF ADE-CQDs on a bacterial biofilm formation, %			10	95.86				[13]
		1	20	100				
			30	100				
		2	10	72.2				
			20	96				
			30	100				
		3	10	34.25				
			20	41				
			30	50				
Minimum inhibitory concentration, µg/mL	^5^ Gram^+^-bacterium					250	350	[16]
	^6^ Gram^−^-bacterium					100	300	
	^7^ Unicellular fungi					350	400	

^1^ Addition time, ^2^ Concentration, ^3^ Hydrothermally-synthesized, ^4^ Infrared-assisted synthesized, ^5^ *Staphylococcus aureus*, ^6^ *Escherichia coli*, ^7^ *Candida albicans*.

## Data Availability

The supporting data for the reported results, including a link to the publicly archived datasets analyzed or generated during the study, can be found under the above-mentioned patents: Justia Patents Search https://patents.justia.com/patent/11060208 (accessed on 17 October 2021).

## Supplementary Materials

The following supporting information can be downloaded at https://www.mdpi.com/article/10.3390/polym15122660/s1, Table S1. Synthesis routes of CQDs from natural macro-precursors and their applications; Table S2. Technical procedures and chemical reagents used for nanocelluloses productions; Table S3. Synthesis of CQDs from synthetic precursors and their applications; Figure S1. Pretreatment of well-plates with histo-blood group antigens (HBGA) present in saliva; Figure S2. Testing binding capacity between viruses like particles (VLPs) and standard antibodies; Figure S3. Testing binding capacity between viruses like particles (VLPs) and passivated-CQDs (EDA-CQDs and EPA-CQDs) using enzyme-linked immunosorbent assay (ELISA) test; Figure S4. Effect of the passivated-quantum dot (P-CQDs): (EDA-CQDs and EPA-CQDs) on viruses like particles (VLPs)-capsid protein using sodium dodecyl sulphate polyacrylamide gel electrophoresis (SDS-PAGE) test and western blotting protocol; Figure S5. Preparation of the gel containing the viruses like particles (VLPs)-passivated-CQDs (GI.1-VLP/EDA-CQDs and GI.1-VLP/EPA-CQDs) used for staining; Figure S6. Preparation of the gel containing the viruses like particles (VLPs)-passivated-CQDs (GI.1-VLP/EDA-CQDs and GI.1-VLP/EPA-CQDs) used for western blotting.

## Nomenclature

**Symbol**	**Definition**	**Symbol**	**Definition**
CNM	Carbon nanomaterials	NCC	Nanocrystalline cellulose
CNT	Carbon nanotubes	n^−^-CQD	Negative carbon quantum dot
COD	Carbon quantum dots	NMR	Nuclear magnetic resonance
DP	Degree of polymerization	NoVs	Noroviruses
DSC	Differential scanning calorimetry	NuPAGE LDS	Gel electrophoresis contains lithium dodecyl sulfate
DTA	Differential thermal analysis	Ox-MWCNTs	Oxidized multiwalled carbon nanotubes
ECL	Electroluminescence	Ox-SWCNTs	Oxidized single walled–carbon nanotubes (Ox-SWCNTs)
ECM	Extracellular matrix (ECM)	PBS	Phosphate-buffered saline
EDA	2,2′-ethylenedioxy-bis-ethylamine	PBST	Phosphate buffered saline with tween 20
ELISA	Enzyme-linked immunosorbent assay	P-CQD	Passivated-carbon quantum dot
EPA	3-ethoxypropylamine	p^+^-CQDs	Positive carbon quantum dot
EPS	Extracellular polymeric substances (EPS)	PL	Photoluminescence
FGY	Fluorescence quantum yield	PS	Particle size
FTIR	Fourier transform infrared spectroscopy	QCE	Quantum confinement effect
GI.1, GII.4	Types of VLPs	ROS	Reactive oxygen species (ROS
GelCode	Blue stain for protein	SCS	Surficial charge status
HBGA	Histo-blood group antigens	SDS-PAGE	Gel electrophoresis containing sodium dodecyl sulphate polyacrylamide
HRP	Horseradish peroxidase	SEM	Scanning electron microscope
KDa	Kilodaltons, a molecular weight unit	TEM	Transmission electron microscope
LCD	Liquid crystal displays	TG	Terminal group of the surface molecule
LED	Light-emitting diodes	TGA	Thermogravimetric analysis
MCC	Microcrystalline cellulose	TMB	3,3′,5,5′-tetramethylbenzidine
MDR	Multidrug resistant	UV	Ultraviolet
MeOH	Methanol	VCN	Viable cell number
MIC	Minimum inhibitory concentration	VLP	Virus-like particles
MOPS SDS	Running buffer: 3-(N-morpholino) propanesulfonic acid	WB	Western blotting
MW	Molecular weight of the surface molecule	XRD	X-ray diffrection
