# Supplementary material for: Nanocellulose-Based Passivated-Carbon Quantum Dots (P-CQDs) for Antimicrobial Applications: A Practical Review
Source: Polymers (Basel). 2023 Jun 12;15(12):2660. doi: 10.3390/polym15122660 (PMC10305638; doi:10.3390/polym15122660)
Supplement: Supplementary file 1 [file polymers-15-02660-s001.zip › polymers-2290760-Supplementary-.pdf]

Supplementary Materials

for

Nanocelluloses-based passivated-carbon quantum dots (P-CQDs) for Antimicrobial Applications: Practical Review

Sherif S. Hindi [1,*]; Jamal S. M. Sabir [2]; Uthman M. Dawoud [3]; Iqbal M. Ismail [4]; Khalid A. Asiry [1]; Zohair M. Mirdad [1]; Kamal A. Abo-Elyousr [1]; Mohamed H. Shiboob [5]; Mohamed A. Gabal [6]; Mona Othman I. Albureikan [7]

[*] Correspondence: shindi@kau.edu.sa; +96-656-676-0086.

This PDF file includes:

Section S1. Natural precursors for synthesis of CQDs.

Table S1. Synthesis routes of CQDs from natural macro-precursors and their applications

Section S2. Synthesis of nanocelluloses, namely microcrystalline cellulose (MCC) and nanocrystalline cellulose (NCC).

Table S2. Technical procedures and chemical reagents used for nanocelluloses productions.

Section S3. Synthetic precursors of CQDs from synthetic precursors.

Table S3. Synthesis of CQDs from synthetic precursors and their applications.

Section S4. Pretreatment of well-plates with histo-blood group antigens.

Figure S1. Pretreatment of well-plates with histo-blood group antigens (HBGA) present in saliva.

Section S5. Binding capacity evaluation between viruses like particles (VLPs) and standard antibodies.

Figure S2. Testing binding capacity between viruses like particles (VLPs) and standard antibodies.

Section S6. Binding capacity evaluation between VLPs and P-CQDs

Figure S3. Testing binding capacity between viruses like particles (VLPs) and passivated-CQDs (EDA-CQDs and EPA-CQDs) using enzyme-linked immunosorbent assay (ELISA) test.

Section S7. Effect of the P-CQDs on viruses like particles (VLPs)-capsid protein

Figure S4. Effect of the passivated-quantum dot (P-CQDs): (EDA-CQDs and EPA-CQDs) on viruses like particles (VLPs)-capsid protein using sodium dodecyl sulphate polyacrylamide gel electrophoresis (SDS-PAGE) test and western blotting protocol.

Section S8. Preparation of the gel-embedded VLPs-treated with P-CQDs for staining.

Figure S5. Preparation of the gel containing the viruses like particles (VLPs)-passivated-CQDs (GI.1-VLP/EDA-CQDs and GI.1-VLP/EPA-CQDs) used for staining.

Section S9. Preparation of the gel-embedded VLPs-treated with CP-CQDs used for western blotting.

Figure S6. Preparation of the gel containing the viruses like particles (VLPs)-passivated-CQDs (GI.1-VLP/EDA-CQDs and GI.1-VLP/EPA-CQDs) used for western blotting.

Section S1. Natural precursors for synthesis of CQDs.

Table S1. Synthesis routes of CQDs from natural macro-precursors and their applications

Synthesis Methods		Precursor	Applications	Reference
	Hydrothermal	Bee pollens	Cellular imaging and catalysis	105
		Sugarcane molasses	Sensoring of Fe3+ and cellular imaging	106
		Pseudo-stem of banana	Sensing Fe+3, Imaging of Hela and MCF-7 cells	107
		Coriander leaves	Sensoring of Fe3+ and cellular imaging	108
		Carrot	Drug delivery	109
		Date kernel	Sensing of drugs and cellular imaging	110
		Papaya juice	Cellular imaging	111
		Saccharum officinarum	Cellular imaging of bacteria and yeast	112
		Sweet potato	Fe+3 sensing and cellular imaging	113
		Walnut shell	Cellular imaging	114
		Water Chestnut and onion	Sensing of Cu (II) and Imaging of Coenzyme A	115
		Apple juice	Imaging of mycobacterium and fungal cells	116
		Chionanthus retusus	Metal ion sensing and imaging of fungal cells	117
		Mangosteen pulp	Sensoring of Fe3+and cellular imaging	118
		Winter melon	Cellular imaging	119
		Strawberry	Fluorescent probes for mercury ions detection	120
		Trapa bispinosa peel	Cellular imaging	121
		Prunus persica	Cellular imaging and oxygen reduction reaction	122
		Prunus mume	Cellular imaging	123
		Garlic	Cellular imaging and free radical scavenging	124
		Onion waste	Sensoring of Fe3+ and cellular imaging	125
		Kidney beans	Cellular imaging	126
		Lemon juice	Optoelectronics and bioimaging	127
		Gelatin	Bioimaging, fluorescent ink	128
		Bergamot	Sensor	129
	Microwave	Bloomed algae	In vitro imaging	130
		Grape seed	Nucleus imaging and pH sensing	131
		Latex	Metal sensing and cellular imaging	132
		Lotus root	Heavy metal ion detection and cellular imaging	133
		Mango leaves	Cellular imaging and Temperature sensors	134
		Tissue paper	Determination of Glutathione	135
	Ultrasonic	Food waste-derived	*In vitro* bioimaging	136
	Ultrasonic & hydrothermal	Lignin biomass	Cellular imaging	137
	Solvothermal	Honey	Sensing Fe^{+3} and imaging of Hep-2 and Hela cells	138
	Carbonization-microwave	Hair	Bioimaging	139
	Microwave radiation	Chitosan derivatives	cell labelling, diagnostics or controlled drug delivery and release systems	140
	Hydrothermal	Microcrystalline cellulose	Producing sensitive probe for metal ion detection in acidic environment.	16
	Pyrolysis	Nanocrystalline cellulose	Fluorescent probes, cellular imaging and temperature sensors	11

[1] References.

Section S2. Synthesis of nanocelluloses, namely microcrystalline cellulose (MCC) and nanocrystalline cellulose (NCC).

Table S2. Technical procedures and chemical reagents used for nanocelluloses' production

Technique	Process	MCC	NCC
		References	
Acid hydrolysis	H_2SO_4	141,142	3,6-9,162
	H_3PO_4	143	143, 163, 164
	HBr	143, 145	145
	HCl	146 147	165
	H_2SO_4 and HCl	148	-
	Formic acid	149	166
	Oxalic Acid	150	167 168
	Maleic acid	151	169, 170
TEMPO Mediated Oxidation	2,2,6,6-tetramethylpiperidinyl1-oxyl (TEMPO)	-	171, 172
Alkali hydrolysis	KOH or NaOH	152	173
Alkali and acidic treatment	H_2SO_4/HCl and NaOH	153,154	-
Steam explosion	Steam and mineral acid	154-157	
	Acid and alkaline treatement then steam treatment	-	174
	Steam only	158	-
Extrusion	Reactive extrusion	159	-
	1st extruded with NaOH, followed by 2nd extrusion with H_2SO_4	160	-
Radiation-enzymatic	Enzymes and electron beam	161	
	Endoglucanase	-	169,170

Section S3. Synthetic precursors of CQDs from synthetic precursors.

Table S3. Synthesis of CQDs from synthetic precursors and their applications [93].

Synthesis Methods	Precursor	Applications	References
Hydrothermal	Ammonium citrate, ethylenediamine	Photocatalysis	173
	Sodium citrate, ethylenediamine	H_2O_2 sensor	175
	Folic acid, ethylene glycol	Hg^{2+} sensor	176
	Citric acid monohydrate, L-cysteine	Bioimaging	177
	Citric acid, linear-structured polyethyleneimine		178
	Citric acid, diethylenetriamine, gadolinium chloride	Bioimaging	179
	Citric acid, cysteamine	Composites, bioimaging	116
	Alanine, ethylenediamine	Biosensing, bioimaging	180
	Citric acid, diethylenetriamine		88
	Citric acid, ethylenediamine	Biosensor, fluorescent ink, composites	181
	Citric acid, $NaBH_4$, ammonia	Sensor	182
	Tetraphenylporphyrin or its metal complex, nitric acid, ethanediamine	Sensor, bioimaging	183
Solvothermal	CCl_4, 1,2 ethylenediamine	pH, Ag^+, $Fe^{3+,}$ H_2O_2 sensor, bioimaging	184
	Anthracite, dimethyl formamide	Composite	185
Microwave	Citric acid, urea, H_2SO_4, H_3PO_4	Screening of oxygen-states in CQDs	14
Microwave pyrolysis	Citric acid, branched polyethyleneimine	In vivo gene delivery	186
Pyrolysis	N-Methyl-2-pyrrolidone, dimethyl-imidazolidinone	Bioimaging, electrocatalysts	187
	Citric acid, branched polyethylenimine	Sensor	188
	L-Glutamic acid	Biological detection	189
Chemical oxidation with acid	Petroleum coke		85
	Coal	Composites	84
	Activated carbon	Sensor	86
Electrochemical	Graphite rods	Catalyst	75
Laser ablation	Carbon target		74
Solid state reaction	Diammonium hydrogen citrate,urea	Sensor, bioimaging, fluorescent ink	190
	Citric acid, dicyandiamide	Sensor	191
Chemical oxidation	Activated carbon	Electrochemiluminescence	192
High-energy ball milling	Activated carbon, KOH	Electrochemiluminescence	83
Electrochemical/ electroanalytical	Amino acids	Bioimaging, sensor	

[1] References

Section S4. Pretreatment of well-plates with histo-blood group antigens.

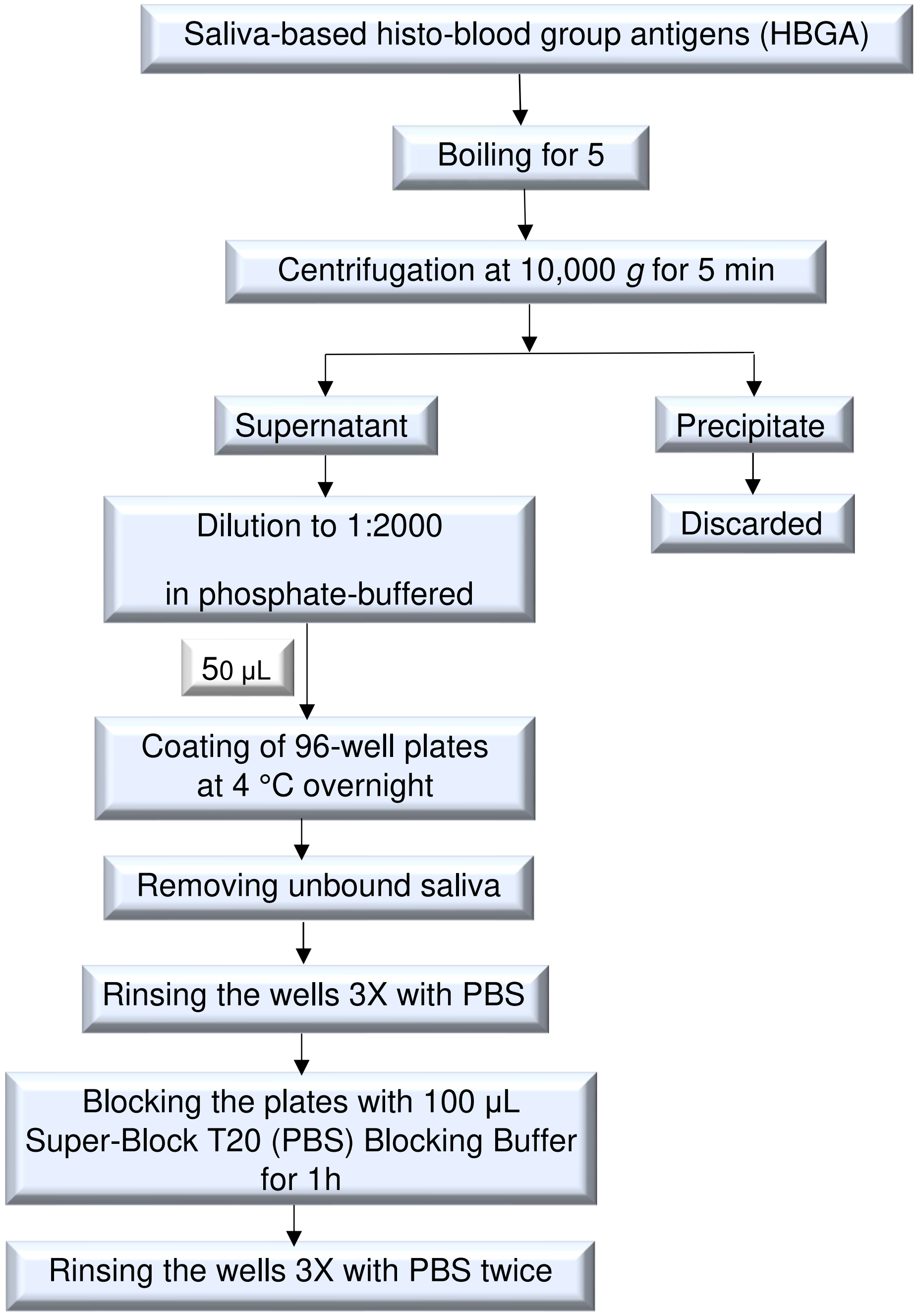

Figure S1. Pretreatment of well-plates with histo-blood group antigens (HBGA) present in saliva.

Section S5. Binding capacity evaluation between viruses like particles (VLPs) and standard antibodies.

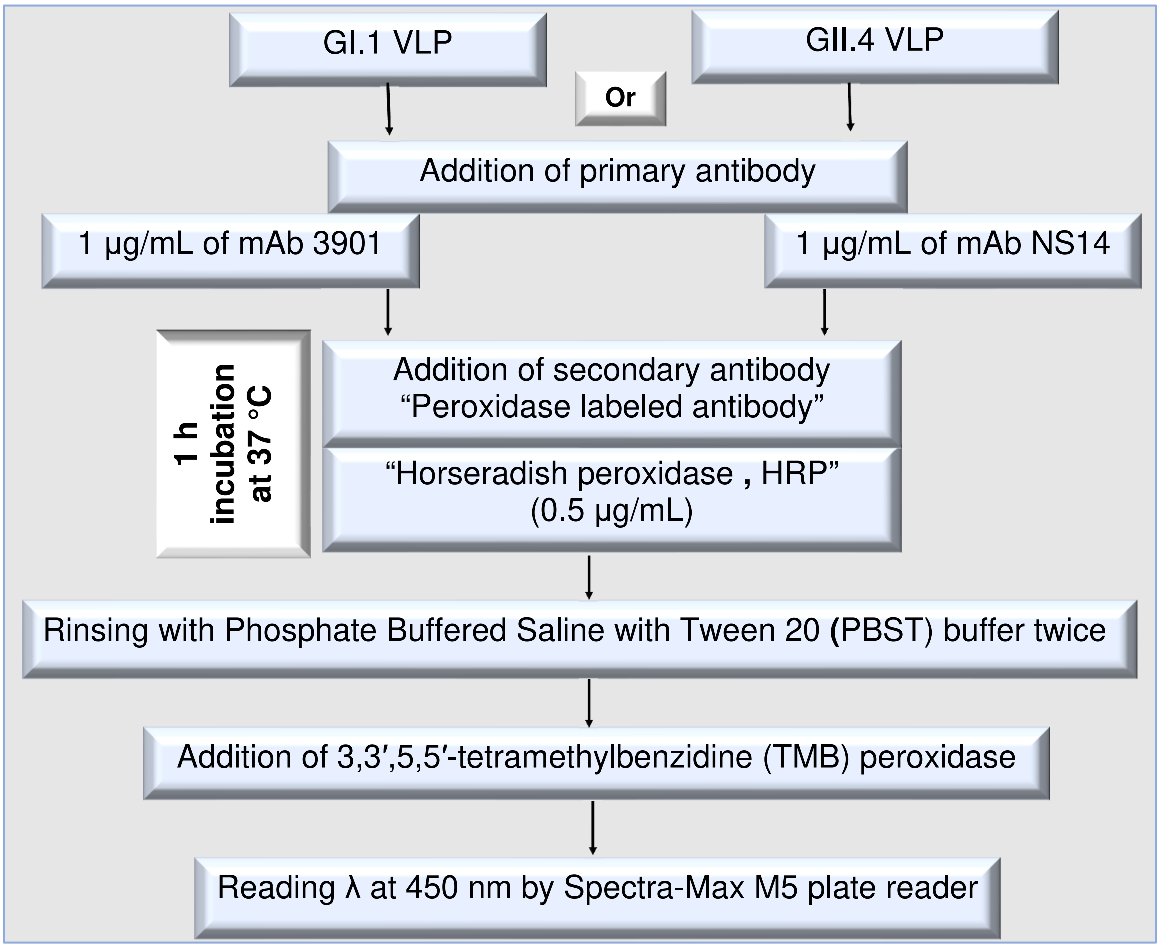

Figure S2. Testing binding capacity between viruses like particles (VLPs) and standard antibodies.

Section S6. Binding capacity evaluation between VLPs and P-CQDs

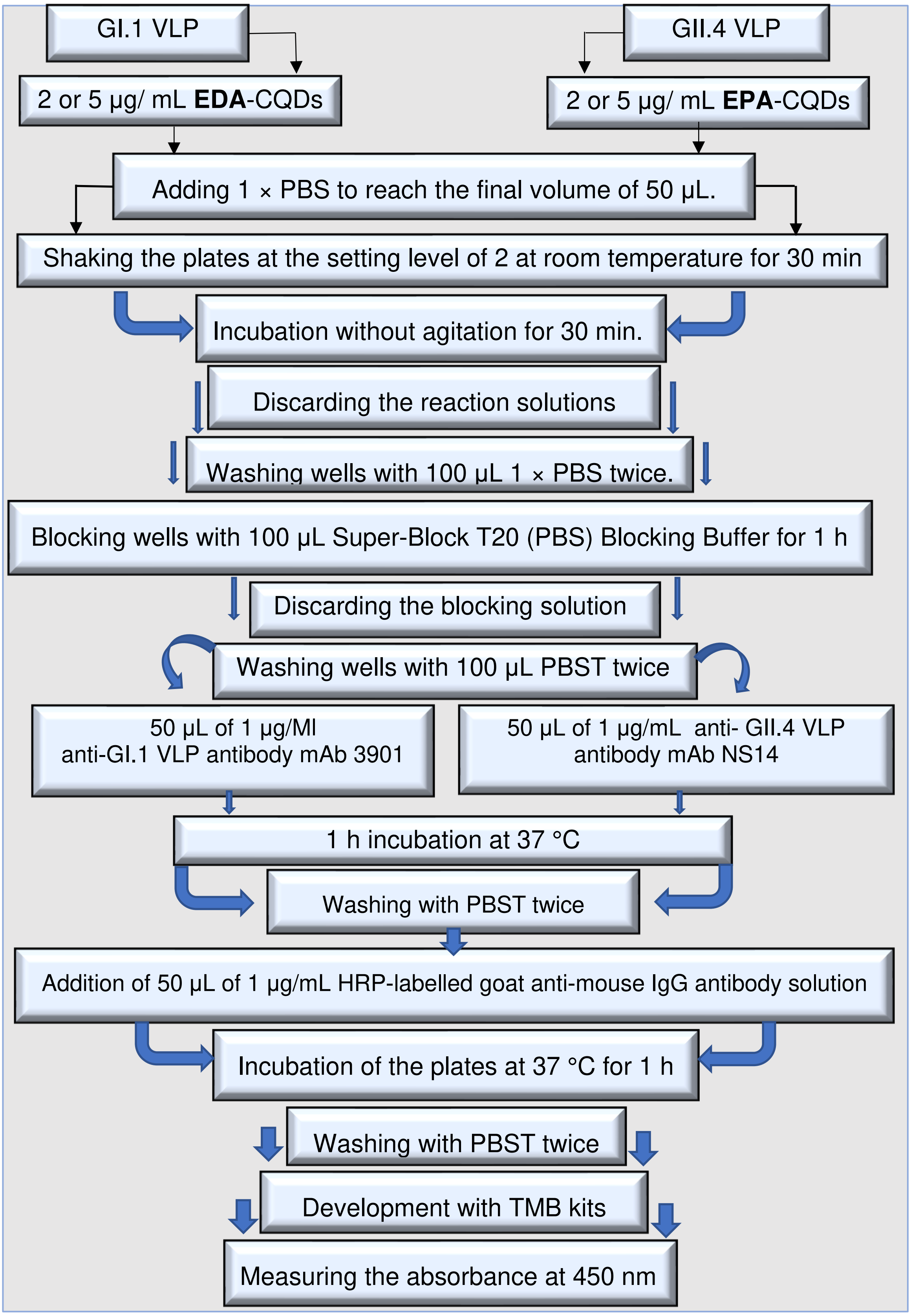

Fig. S3. Testing binding capacity between viruses like particles (VLPs) and passivated-CQDs (EDA-CQDs and EPA-CQDs) using enzyme-linked immunosorbent assay (ELISA) test.

Section S7. Effect of the P-CQDs on viruses like particles (VLPs)-capsid protein

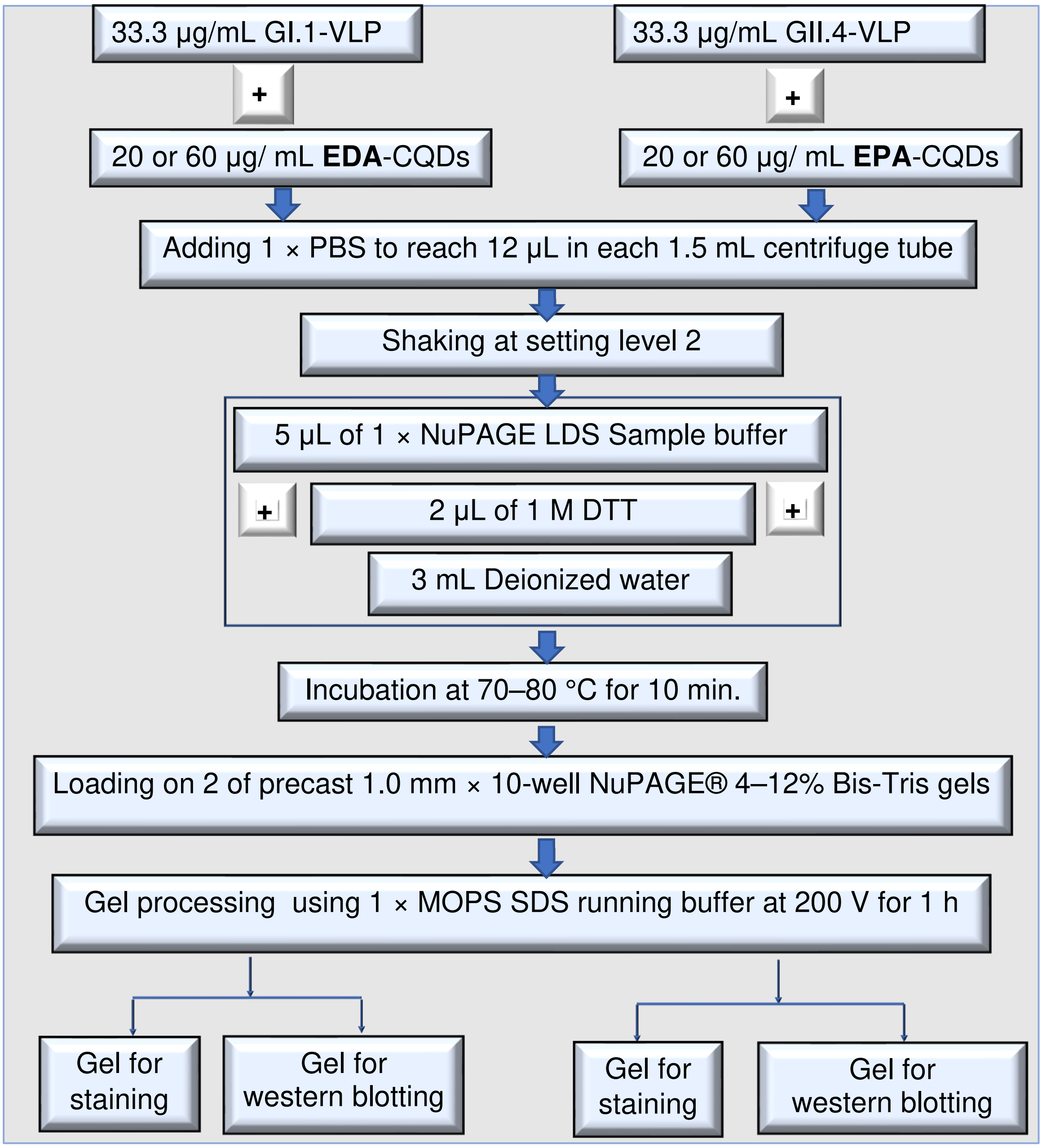

Figure S4. Effect of the passivated-quantum dot (P-CQDs): (EDA-CQDs and EPA-CQDs) on viruses like particles (VLPs)-capsid protein using sodium dodecyl sulphate polyacrylamide gel electrophoresis (SDS-PAGE) test and western blotting protocol.

Section S8. Preparation of the gel-embedded VLPs-treated with P-CQDs for staining.

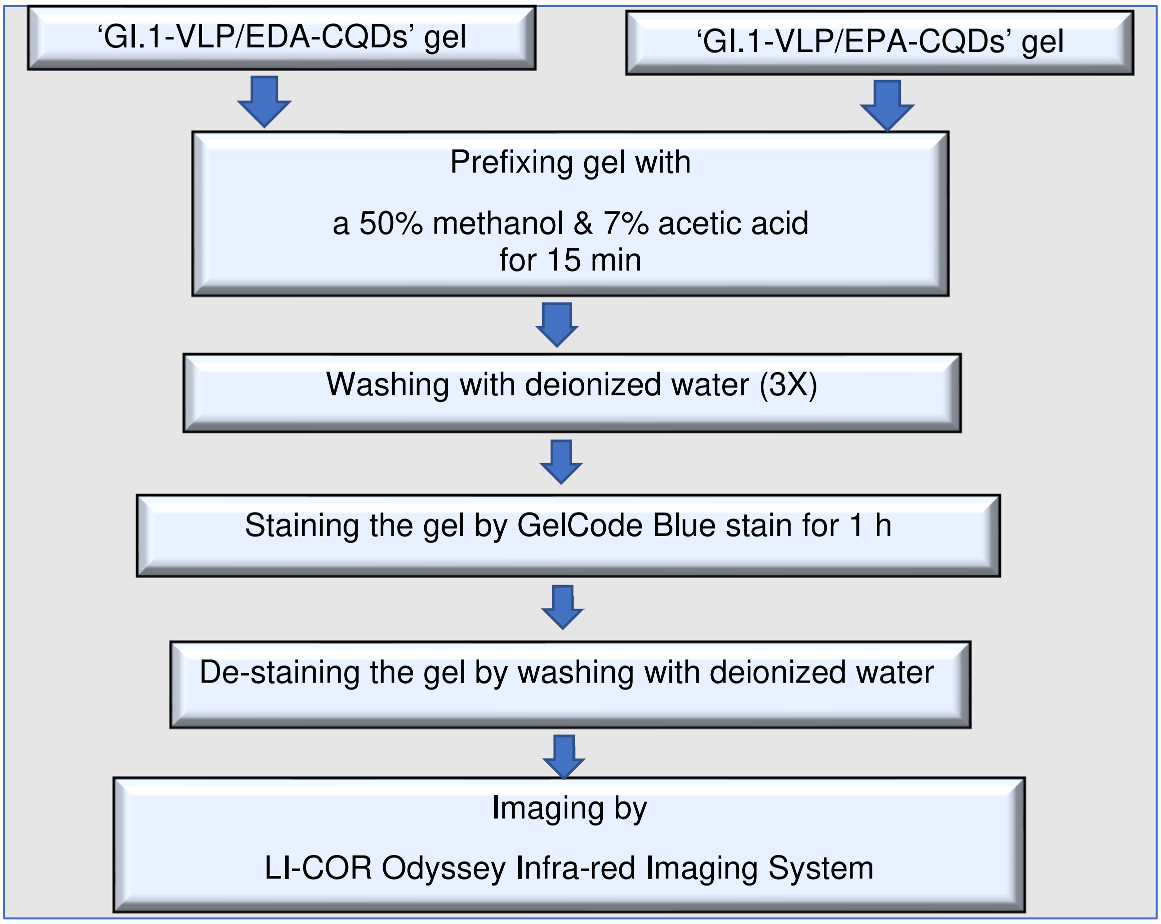

Figure S5. Preparation of the gel containing the viruses like particles (VLPs)-passivated-CQDs (GI.1-VLP/EDA-CQDs and GI.1-VLP/EPA-CQDs) used for staining.

Section S9. Preparation of the gel-embedded VLPs-treated with CP-CQDs used for western blotting.

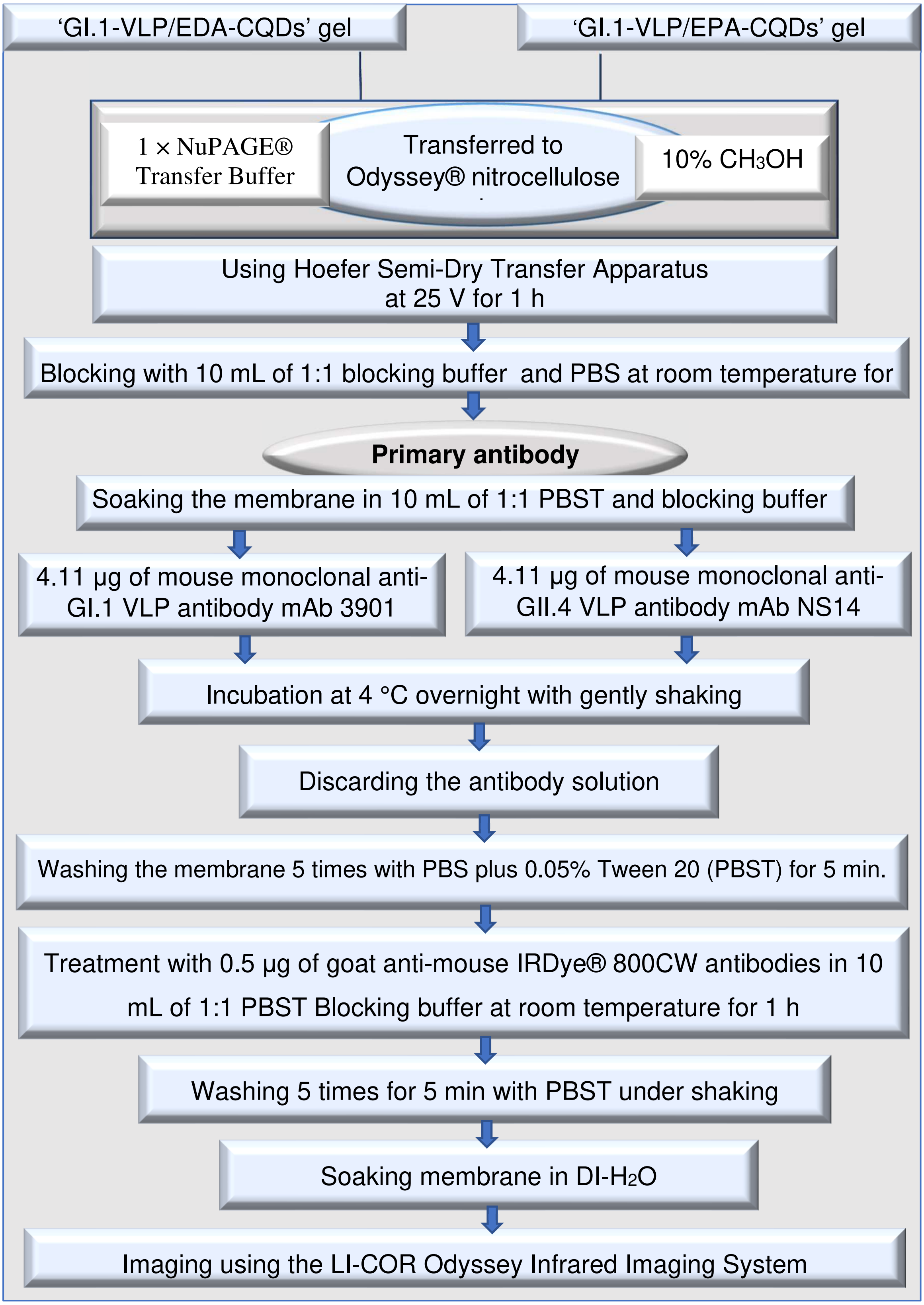

Figure S6. Preparation of the gel containing the viruses like particles (VLPs)-passivated-CQDs (GI.1-VLP/EDA-CQDs and GI.1-VLP/EPA-CQDs) used for western blotting.
